# Stress/depression across the COVID-19 pandemic in Denmark

**DOI:** 10.1186/s12889-023-15129-5

**Published:** 2023-01-25

**Authors:** Marcelo Cardona, Lars H. Andersen, Peter Fallesen, Tim A. Bruckner

**Affiliations:** 1 ROCKWOOL Foundation, Ny Kongensgade 6, 1472 Copenhagen C, Denmark; 2 Swedish Institute for Social Research, Stockholm University, 106-91 Stockholm, Sweden; 3 Public Health, University of California, 653 E. Peltason Dr, 92697-3957 Irvine, CA USA

**Keywords:** Mental health, Stress/depression, WHO-5, COVID-19, Denmark, Gender, Longitudinal data

## Abstract

**Background:**

Global estimates suggest strained mental health during the first year of the COVID-19 pandemic, but the lack of nationally representative and longitudinal data with clinically validated measures limits knowledge longer into the pandemic.

**Methods:**

Data from 10 rounds of nationally representative surveys from Denmark tracked trends in risk of stress/depression from just before the first lockdown and through to April 2022. We focused on age groups and men and women in different living arrangements and controlled for seasonality in mental health that could otherwise be spuriously related to pandemic intensity.

**Results:**

Prior to first lockdown, we observed a “parent gap”, which closed with the first lockdown. Instead, a gender gap materialized, with women experiencing higher risks than men—and higher than levels predating first lockdown. Older respondents (+ 70 years) experienced increasing risks of stress/depression early in the pandemic, while all other groups experienced decreases. But longer into the pandemic, risks increased for all age groups and reached (and sometimes exceeded) levels from before first lockdown.

**Conclusion:**

Denmark had low infection rates throughout most of the pandemic, low mortality rates across the entire pandemic, and offered financial aid packages to curb financial strains. Despite this circumstance, initial improvements to mental health during the first lockdown in Denmark were short-lived. Two years of pandemic societal restrictions correspond with deteriorating mental health, as well as a change from a parenthood gap in mental health before first lockdown to a gender gap two years into the pandemic.

**Supplementary Information:**

The online version contains supplementary material available at 10.1186/s12889-023-15129-5.

## Introduction

The Global Burden of Disease (GBD) Study estimates that global prevalence of major depressive symptoms increased by 27.6% during the first year of the COVID-19 pandemic, from 2,470 to 3,153 per 100,000 [1]. It also highlights variation across groups and reviewed studies, however, with for example women experiencing 29.8% increase, 24.0% for men [1, 2]. Other research finds more divergent results than the GBD [3–9], even documenting mental health *benefits* immediately following lockdowns [5, 6]. Moreover, no previous study documented the time-course of population mental health across more than the first 1½ years of the pandemic.

A recent study used data from late-April 2020 to late-June 2021 covering 15 countries to conclude that stricter policies and case and death rates were associated with worse mental health, although the association was weak and driven by adherence to physical distancing guidelines [10]. But level of analysis – countries – could mask heterogenous impacts on groups within countries. In Denmark, the context of our study, a positive correlation between mental health problems and pandemic intensity was found using panel data, with some heterogeneity across age [7, 11–14]. But studies did not establish a pre-pandemic/pre-lockdown baseline, except for Würtzen et al. [15] and Thygesen et al. [16] who measured baselines 1–4 years prior. However, these studies either lacked a pre-lockdown baseline measure, or did not consider variation in mental health across the course of the pandemic. Furthermore, few existing studies rely on longitudinal, nationally representative data with validated clinical measures of mental health. Also important, none of the studies consider the potentially important role of weather and seasonality in mental health [11, 17, 18], which may correlate with pandemic intensity.

We present results for the development in the risk of stress/depression for different age groups, across gender, and people in different living arrangements (as defined from whether one has children living at home) while controlling for seasonality across more than two pandemic years in Denmark. The age and gender gradients in COVID-19 mortality risk [19] could lead to differences in mental strain across those characteristics. Similarly, gendered division of home schooling and housework [20, 21] may have imposed different constraints across gender and living arrangements.

We used 10 rounds of combined longitudinal and cross-sectional survey data from just before the first lockdown in Denmark in March of 2020 and through to April of 2022. Surveys were nationally representative and contained validated clinical measures of mental health. Our study contributes to research on the mental health consequences of social and health restrictions during the pandemic, but also provides a representative country profile for how mental health unfolded across the pandemic for different age groups and people in different living arrangements. We also, unlike earlier work, control for seasonality and analyse the importance of local infection rates in a country with low infection rates through the first waves and little to no excess mortality that still saw substantial social barriers because of the pandemic [6, 22–25].

## Methods

### Study design

This population survey included respondents to one or more of 10 surveys carried out in Denmark during March 2020 to March 2022. First data collection (random sample from the adult population), performed by Statistics Denmark on behalf of the Capital Region of Denmark’s Mental Health Services (CRDMHS), was during March-April 2020, coincidentally overlapping with the first societal lockdown in Denmark on March 11, 2020, thus allowing a pre-lockdown baseline. CRDMHS originally aimed to survey the Danish adult population on multiple mental health dimensions including risk indicators for stress/depression [the World Health Organization Five Well-being Index (WHO5), for example, to which we return]. Respondents were contacted via their digital post-box, which is mandatory for official communication with public authorities. Statistics Denmark recontacted respondents in July and November of 2020, in March and November of 2021, and in March of 2022, to produce a longitudinal dataset. To observe the potential threat of attrition for our conclusions, we also – again collaborating with CRDMHS – made Statistics Denmark reproduce parts of the initial survey on new random draws from the population in September of 2020, March and September of 2021, and March of 2022.

Statistics Denmark holds individual level data on the full population based of administrative registries [26], offering several benefits. First, it offers straightforward drawing of random samples from the population of specific ages, as all citizens have individual identifiers and are recorded with date of birth (the official nature of the data precludes undocumented immigrants from participation, and our results do not apply to this group). Second, it offers analyses of attrition, which document imbalances between samples and the population. Third, it offers population weights to consider said attrition. Statistics Denmark provided these population weights (except for the July 2020 data, to which we return). Surveys were answered using computer assisted web interviews, and informed consent to participate from the study participants was obtained prior to responding. Non-responders received prompts via messages in their digital post-box. To reduce attrition in the longitudinal surveys, gift certificates were randomly awarded to respondents.

### Procedures

To assess the time-course in the risk of stress/depression across the pandemic in Denmark, we relied on survey answers to the WHO5, a widely validated clinical tool that screens for the risk of developing stress and depression [27]. Five items ask about wellbeing over the previous two weeks, measured between 0 and 100, with each item contributing 0–20 points. 0 indicates the most severe depressive symptoms and 100 indicates no symptoms. The CRDMHS provided detailed information on the WHO5, including the specific items (information available in several languages [28]). For Denmark, the adult population norm is 70 [29]. We used the binary threshold (WHO5 < 50), which is also the threshold used in clinical settings [27], for whether a person is at risk of stress/depression for our main specification. One challenge is that the items in the WHO5 inquire about a two-week recall period, as this period may overlap with changes in societal restrictions, most notably with lockdowns. Prior research, however, has not found it to be the case [6].

As explanatory variable, we focused on survey round to track the risk of stress/depression across the pandemic. Since the GBD study [2] and a recent report from the European Foundation for the Improvement of Living and Working Conditions [20] emphasize potentially heterogenous impacts across age and gender, we focused also on age groups (six groups: 18–29, 30–39, …,70–79) and on men and women in different living arrangements (children younger than 18 years living at home or not; also see [6]). Respondents could switch categories on these variables across the survey rounds.

To control for weather (and hence seasonality [17, 18]), we used daily measures of average temperature and hours of sunshine from the Danish Meteorological Institute. For each response date in our data, we added these weather measures on the day before.

Danish infection rates were low through the first waves of the pandemic, mortality rates have been low throughout the pandemic, and governmental aid packages have helped curb most economic impacts of the pandemic on people’s lives. Local threat of infection, however, could still matter for wellbeing. To enable the examination of this mechanism in a robustness analysis (to which we return), we merged onto the survey data publicly available official daily municipal-level test data [30].

### Statistical analysis

We first described the data in terms of number of invited respondents, response rates, and data collection periods, and we summarized survey respondents relative to population on key characteristics. The first survey round coincided with the first lockdown in Denmark, as mentioned, wherefore we split it into pre/post lockdown data to obtain pre-lockdown comparison estimates. Previous research [6] has documented no problems related to inference in this procedure, as no selection can be observed in who responded prior to and during lockdown; documentation we replicated. Because some respondents appeared in the (longitudinal) data more than once, we clustered standard errors using individual identifiers. We applied the population weights provided by Statistics Denmark. Because we received population weights per survey round the weights did not account for our splitting of the first survey round into pre/post lockdown periods (but due to no observed selection in pre/post respondents this is likely inconsequential). Also, because we did not receive population weights for the July 2020 longitudinal data, we here relied on weights from March 2020. These issues with imprecise population weights for some respondents are likely unimportant for our results as use of weights did not matter for our results, see robustness analyses.

Two linear regressions with controls evaluated the risk of stress/depression for different age groups and by gender in different living arrangements. Both regressions had as the outcome variable the binary indicator of scoring below the clinical threshold value for being at risk of stress or depression (WHO5 < 50). In both regressions we controlled for weather (proxy for seasonality). In the first regression, we added survey round by age group fixed effects, providing a point estimate for each age group in each survey round. In the second regression, we added survey round by gender by living arrangement fixed effects, again providing for each survey round separate point estimates for men and women in different living arrangements.

We present results as plots of the predicted outcome and 95% confidence intervals by comparison groups across the survey rounds. To illustrate how these results evolved with the pandemic and the policies imposed as a reaction to it, we also graph the total daily number of confirmed infections and mark the imposing and lifting of societal lockdowns in Denmark. As supplementary results, we modelled point estimates tested against the within-survey round baseline for the age group 18–29 years and men without children at home, respectively, to allow for inter-group comparisons in trends rather than estimated risks for stress/depression.

We conducted several robustness checks (online appendix). First, we evaluated whether results were sensitive to our focus on the clinically relevant version of the WHO5 (WHO5 < 50) by replicating analyses using its continuous version. Second, to evaluate whether the natural aging of returning respondents in the longitudinal data invalidate our results, we reran models while fixing respondents to the age group in which they initially entered the data. Third, to evaluate sensitivity to population weights, we reproduced results using unweighted data. Last, to observe whether changes in the local threat of infection influenced the risk of stress/depression, we analysed whether the risk correlated meaningfully with local infection rates. We further detail this last robustness check in the online appendix.

## Results

Table 1 describes the 10 survey rounds including collection dates and response rates. Our total data consisted of 22,858 responses from 15,685 unique persons (2,821 respondents in Round 1 also provided 7,173 responses to later longitudinal surveys). Overall response rate was 32.4% (on average 26.7% for cross-sectional data).

**Table 1:** Description of survey rounds

Survey Round	Sample Size	Response Rate	Number of responses	Sample Type	Collection period
1	8,300	0.340	2,821	Longitudinal (initial)	March 2 – April 13, 2020
2	2,818	0.550	1,549	Longitudinal	July 2 – August 01, 2020
3	8,200	0.352	2,883	Cross-sectional	September 01 – September 30, 2020
4	2,818	0.579	1,632	Longitudinal	November 04 – November 30, 2020
5	13,360	0.223	2,978	Cross-sectional	March 03 – April 24, 2021
6	2,802	0.550	1,541	Longitudinal	March 03 – April 24, 2021
7	13,375	0.286	3,821	Cross-sectional	September 14 – November 26, 2021
8	2,796	0.541	1,513	Longitudinal	November 12 – December 30, 2021
9	13,325	0.239	3,182	Cross-sectional	March 01 – April 18, 2022
10	2,781	0.337	938	Longitudinal	March 01 – April 18, 2022
Total	70,575	0.324	22,858	N/A	March 2, 2020 – April 18, 2022

Note: All data collected by Statistics Denmark with the approval of the Capital Region of Denmark’s Mental Health Services and under informed consent from the participants. Statistics Denmark randomly selected respondents (using unique personal identification numbers) from the full population aged 18–79. All respondents to Survey Round 1 were contacted on March 1st, 2020, via their official and mandatory Danish digital post-box. In subsequent rounds of the longitudinal sample, respondents to the previous round of the longitudinal sample were recontacted (using the same method), which explains the declining number of responses for the longitudinal sample. Cross-sectional samples each represent a new random draw from the population performed by Statistics Denmark; again, all respondents were contacted via the digital post-box just prior to the opening of the survey round. The total sample size reported in the bottom row of the table hence includes returning respondents; the number of unique respondents was 15,685. Decline in sample size for longitudinal sample due to outmigration and mortality.

Respondents predominantly were older than 50 years of age, in relationships, and had no children living at home. Across survey rounds, 25–35% of the sample had children living at home (Table A1). But none of the characteristics were unbalanced in any systematic way across surveys and population (Table A1 compares means and standard deviations to the population). In addition, Table A2 documents no sizeable or systematic changes to the composition of the longitudinal data because of attrition, and Table A3 compares respondents pre/post first lockdown to document no selection in response patterns across the first lockdown.

**Fig. 1:**
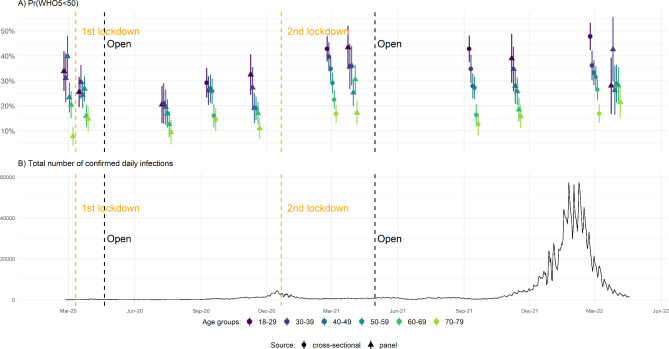
Risk of Stress/Depression Across Age Groups and Number of Total Daily Confirmed Cases of Infection, from March 2020 to April 2022 in Denmark. (Note: For details on data collection periods, survey response rates, and numbers of observations, see Table 1)

Panel A of Fig. 1 shows the estimated risk of stress/depression for age groups (controlling for seasonality). Panel B shows the total daily confirmed infection numbers in Denmark. We split the first survey round by the beginning of the first lockdown to obtain pre-lockdown comparison estimates (hence 11 sets of point estimates in Fig. 1). Table A4 reports estimates tested against the baseline for the age group 18–29 years within survey rounds.

Results documented declines in the share at risk for stress/depression during the early stages of the pandemic and shortly after Denmark’s first lockdown on March 11th 2020, replicating previous research [6]. We observed this pattern for most of the age groups, except for the oldest respondents (70–79 years), for whom we observed increased risks (they still had the lowest absolute risks, however; increase from 7.8% [95% CI: 4.1–11.6%] to 14.8% [95% CI: 9.8–19.8%], p = 0.024, Table A5). The new and lower plateau for the risks of stress/depression remained visible through July 2020, where most lockdown restrictions had been lifted, and infection and mortality rates were low.

Late-August 2020 experienced the onset of the second wave of infections and the risk of stress/depression increased for all age groups but the oldest. Across the fall of 2020 new government restrictions were reintroduced. Most importantly, Denmark had a second lockdown between December 16th 2020 and May 18th 2021. Formal lockdown was not as long as the dates imply, as society gradually reopened from March 1st, 2021. By early 2021, during the second lockdown, all age groups had increased to above (or at least at) pre-pandemic levels. Most notably, 18–29-year-olds estimated risk now reached 42.8% (95% CI: 37.7–47.8%, Table A5), compared to 25.5% (95% CI: 19.5–31.6%, Table A5) during first lockdown. Oldest respondents’ level now more than doubled relative to before first lockdown (16.8% [95% CI: 13.3–20.4%] vs. 7.8% [95% CI: 4.1–11.6%], p < 0.001, Table A5). The overall increase and age-related pattern from before the pandemic in stress/depression thus re-emerged more strongly up until this point in the pandemic.

Risks for stress/depression declined slightly overall following the lifting of the second lockdown. We again observed a clear age patterning: risks had decreased substantially by Autumn 2021 for anyone older than 60 years, whereas they remained almost as high as during the second lockdown for respondents younger than 40 years.

At the end of 2021, the Omicron virus variant surged confirmed infections. The total daily number of confirmed new infections rose from few in October and November to over 50,000 in January and February. From this point on, infections decreased. During this period, we had three survey rounds. Results from these rounds indicate convergence in risks in late 2021 when infections rates increased, implying that older respondents saw increasing risks while younger saw stable or decreasing risks.

Just after the infection peak in early 2022, however, risks had again increased across all age groups. At this point, results from cross-sectional data and longitudinal data differ somewhat (most notably for 18-29-year-olds: 47.8% in cross-sectional data [95% CI: 42.3–53.2%], 28.0% in longitudinal data [95% CI: 16.7–39.3%], p = 0.002; see Table A5). Results from the longitudinal data suggest less variability across age groups than from the cross-sectional data. Considering the width of confidence intervals for estimates from the March 2022 longitudinal data, differences may pertain to increased attrition in the longitudinal data between November 2021 and March 2022.

**Fig. 2:**
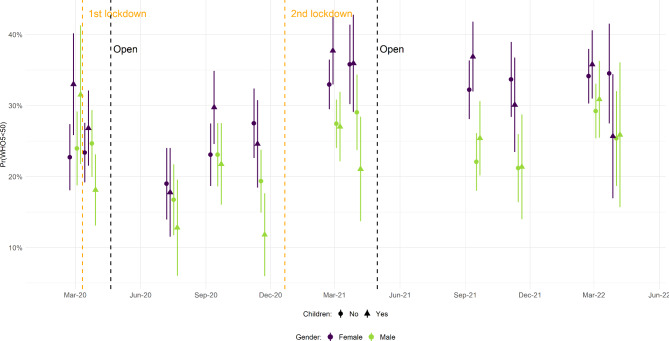
Development in Risk of Stress/Depression Across Gender and Parenthood over the Entire Pandemic in Denmark. (Note: For details on data collection periods, survey response rates, and numbers of observations, see Table 1)

Figure 2 reports the findings across gender and living arrangements, again controlling for seasonality (point estimates in Table A6-A7). The risk of stress/depression improved during the first lockdown and into summer of 2020, which was most pronounced for men and women who had children living at home (and who had the highest pre-lockdown risks). This finding confirmed previous research [6]. From September 2020 and onwards, however, risks increased in a clearly gendered way, mirroring the well-documented gender gap in mental health [31]. Whereas men, irrespective of living arrangement, increased to similar risk as before the first lockdown, women, also irrespective of living arrangement, now faced higher risks. This gender difference persisted throughout the rest of the period. Here, women’s risks only fell below 30% in one out of 12 estimates (women with children at home in March 2022) whereas men’s risks only exceeded that level in one out of 12 estimates (men with children at home in March 2022). Evaluated across all survey rounds from the introduction of the second lockdown (when the gender difference was evident) shows that the gender gap in the risk of stress/depression during this period was 10.2% points (p < 0.001). Importantly, this difference was found irrespective of living arrangements.

### Summary of robustness analyses

The results from our robustness analyses uniformly confirmed that the transformation of our outcome variable (Tables A8 and A9), the aging of returning respondents (Tables A10), the application of population weights (Figures A1 and A2), and the infection rate in the respondents’ local area (Appendix B) did not substantially affect our main conclusions.

## Discussion

Few studies on the consequences of the COVID-19 pandemic for mental health leverage longitudinal, nationally representative data with validated clinical measures of mental health. Our study leveraged all three of these data features, relying on both longitudinal and cross-sectional data from 10 survey rounds from Denmark across the first two pandemic years, which contained measures of the WHO5 Well-being Index. Whereas a brief “honeymoon effect” in Denmark occurred shortly after the onset of the first lockdown, with time the risks of stress/depression returned to (and for some groups exceeded) pre-pandemic levels. This pattern differs markedly by gender, with women showing elevated risk of stress/depression as the pandemic persisted.

Our results contribute to knowledge on which subgroups disproportionately suffer the mental health burden of the pandemic [1, 5, 9, 11, 32–34]. The oldest respondents—70–79 years-old who are most at risk for adverse consequences of a COVID-19 infection—represent the only age-group with a significant increase in the risk of developing stress/depression at the onset of the pandemic, corroborating findings from an age-specific lockdown in Turkey [35]. This development should cause concern, as previously expressed [36]. But importantly, this group had the lowest risk of stress/depression, implying that although they experienced wellbeing declines during the pandemic, their risk was lower than for other age groups.

All other age groups showed lower than expected levels of stress/depression during the first societal lockdown in Denmark, which is consistent with prior research and mirrors the mental health “honeymoon effect” observed in studies of disasters [37]. These age groups, however, then slowly reported higher risks of stress/depression, with the largest increases (and greatest overall risk) occurring among the youngest.

Looking at gender and living arrangements, it is striking how following the first lockdown, we observed a closure of a pre-pandemic ‘parent gap’ in mental health, but as the pandemic persisted, the parent gap remained closed while a ‘gender gap’ opened in its stead. Women suffered higher risks of stress/depression throughout the remainder of our data period.

Our findings are broadly consistent with international results from the GBD Study [2]. One finding unique to our study is the substitution of a parent gap for a gender gap in the risk of stress/depression. For women, and mothers in particular, COVID-19 studies point to unequal division of care responsibilities and housework as a likely driver of the increasing gender gradient in mental health [20, 21, 33]. Our finding could reflect this return to more traditionalist gender roles during the pandemic even in gender-egalitarian Denmark. The suggestion is, however, interpretive and in need of future testing.

The mental health toll for young adults may have arisen from social distancing requirements, as was recently indicated in multi-country research [10]. This explanation, however, also remains speculative and could not be tested with our data. We therefore encourage future research to test the importance of (changing) gender roles during the pandemic, the role of social distancing requirements especially for young people’s mental health, and the causes of elevated sensitivity of younger adults to COVID-19 related stressors, as well as of their generally greater self-reported risk of stress/depression.

Limitations to our study include low N for some survey rounds, causing wide confidence intervals. Also, attrition is a core challenge to longitudinal studies. We did, however, manage to keep around half of the original respondents through our longitudinal surveys, except the last round where response rate was one in three. A core benefit of Statistics Denmark handling the data collection was the provision of population weights, and attrition is thus not likely to drive our results. Another limitation concerns the role of infection, as we lacked individual level information on infection and, also importantly, knowing infected family members. This limitation precludes using our results to infer any individual-level stress/depression response to coping with COVID-19 infection in the household. Our results, instead, should be interpreted as population-level averages for each time point. Another limitation concerns unobserved confounding, and although we, as the first study that we know of, controlled for seasonality, associations between survey round and the risk of stress/depression could still be influenced by other time sensitive features. Last, we cannot rule out that estimates for early Spring 2022 also reflect the critical situation of rising inflation rates and the war in Ukraine, which may have additionally strained respondents’ wellbeing.

## Conclusion

The Denmark country profile in mental health across age groups and gender in different living arrangements shows that although older people experienced decreased mental health in the early stages of the pandemic (which persisted throughout), they generally had the lowest risk of stress/depression. Young people and parents (both fathers and mothers) experienced an initial mental health improvement during first lockdown. Young people then experienced longer term deterioration of mental health. The parent gap in mental health remained closed while a gender gap emerged during the pandemic. This country profile is likely driven by societal restrictions, rather than infection rates or financial worry caused by the pandemic, as Denmark managed to minimize other facets of the pandemic relative to other countries.

## Electronic supplementary material

Below is the link to the electronic supplementary material.

Supplementary Material 1

## Data Availability

The data that support the findings of this study are available from [Statistics Denmark and the ROCKWOOL Foundation, but restrictions apply to the availability of these data, which were used under license for the current study, and so are not publicly available. The data cannot be made available outside the hosted research servers at Statistics Denmark. University-based and private Danish scientific organisations can be authorised to work with data within Statistics Denmark. Such organisations can provide access to individual scientists inside and outside of Denmark. Requests for data may be sent to Statistics Denmark: http://www.dst.dk/en/OmDS/organisation/TelefonbogOrg. aspx?kontor = 13&tlfbogsort = sektion or the Danish Data Protection Agency: https://www.datatilsynet.dk/english/the-danish-data-protection-agency/contact/. The authors document and make available all code needed to reproduce the findings in the study (see Appendix C), and the corresponding author can be contacted for questions regarding data access.
