# Supplementary material for: Stress/depression across the COVID-19 pandemic in Denmark
Source: BMC Public Health. 2023 Jan 25;23:169. doi: 10.1186/s12889-023-15129-5 (PMC9875528; doi:10.1186/s12889-023-15129-5)
Supplement: Supplementary file 1 — Supplementary Material 1 [file 12889_2023_15129_MOESM1_ESM.docx]

**APPENDICES**

**Appendix A**

Table A1: Comparisons of means and standard deviations on key variables in cross-sectional surveys and population.

|  | Variable |  |  |  |  |  |  |  |  |  |
| --- | --- | --- | --- | --- | --- | --- | --- | --- | --- | --- |
| Survey round and statistic | 18-29 years | 30-39 years | 40-49 years | 50-59 years | 60-69 years | 70-79 years | Children at home | In a relationship | Female | N |
| **Round 1** |  |  |  |  |  |  |  |  |  |  |
| Survey mean | 0.136 | 0.110 | 0.158 | 0.224 | 0.207 | 0.164 | 0.325 | 0.700 | 0.551 | 2,821 |
| Survey std.dev. | 0.343 | 0.313 | 0.365 | 0.417 | 0.405 | 0.370 | 0.469 | 0.458 | 0.497 |  |
| Population mean | 0.207 | 0.156 | 0.173 | 0.184 | 0.152 | 0.128 | 0.371 | 0.650 | 0.502 | 4,318,684 |
| Population std.dev. | 0.405 | 0.363 | 0.379 | 0.387 | 0.359 | 0.334 | 0.483 | 0.477 | 0.500 |  |
| **Round 3** |  |  |  |  |  |  |  |  |  |  |
| Survey mean | 0.127 | 0.128 | 0.151 | 0.215 | 0.219** | 0.160 | 0.335 | 0.697 | 0.557 | 2,883 |
| Survey std.dev. | 0.333 | 0.334 | 0.358 | 0.411 | 0.414 | 0.367 | 0.472 | 0.460 | 0.497 |  |
| Population mean | 0.207 | 0.156 | 0.173 | 0.184 | 0.152 | 0.128 | 0.371 | 0.650 | 0.502 | 4,318,684 |
| Population std.dev. | 0.405 | 0.363 | 0.379 | 0.387 | 0.359 | 0.334 | 0.483 | 0.477 | 0.500 |  |
| **Round 5** |  |  |  |  |  |  |  |  |  |  |
| Survey mean | 0.139 | 0.105 | 0.138*** | 0.208 | 0.220** | 0.189*** | 0.299* | 0.686 | 0.539 | 2,978 |
| Survey std.dev. | 0.346 | 0.307 | 0.345 | 0.406 | 0.414 | 0.392 | 0.458 | 0.464 | 0.499 |  |
| Population mean | 0.207 | 0.156 | 0.173 | 0.184 | 0.152 | 0.128 | 0.371 | 0.650 | 0.502 | 4,318,684 |
| Population std.dev. | 0.405 | 0.363 | 0.379 | 0.387 | 0.359 | 0.334 | 0.483 | 0.477 | 0.500 |  |
| **Round 7** |  |  |  |  |  |  |  |  |  |  |
| Survey mean | 0.144 | 0.114* | 0.142* | 0.209 | 0.215* | 0.175 | 0.304 | 0.697 | 0.554 | 3,821 |
| Survey std.dev. | 0.352 | 0.318 | 0.349 | 0.407 | 0.411 | 0.380 | 0.460 | 0.460 | 0.497 |  |
| Population mean | 0.207 | 0.156 | 0.173 | 0.184 | 0.152 | 0.128 | 0.371 | 0.650 | 0.502 | 4,318,684 |
| Population std.dev. | 0.405 | 0.363 | 0.379 | 0.387 | 0.359 | 0.334 | 0.483 | 0.477 | 0.500 |  |
| **Round 9** |  |  |  |  |  |  |  |  |  |  |
| Survey mean | 0.127 | 0.096* | 0.145 | 0.203 | 0.224* | 0.206 | 0.291 | 0.681 | 0.557 | 3,182 |
| Survey std.dev. | 0.333 | 0.294 | 0.352 | 0.403 | 0.417 | 0.404 | 0.454 | 0.466 | 0.497 |  |
| Population mean | 0.207 | 0.156 | 0.173 | 0.184 | 0.152 | 0.128 | 0.371 | 0.650 | 0.502 | 4,318,684 |
| Population std.dev. | 0.405 | 0.363 | 0.379 | 0.387 | 0.359 | 0.334 | 0.483 | 0.477 | 0.500 |  |

*Notes:* Significance stars refer to tests of survey mean and standard deviation (std.dev.) against the population mean and standard deviation within rounds.
* p < 0.05; ** p < 0.01; *** p < 0.001 (two-tailed tests).

Table A2: Comparisons of means and standard deviations on key variables in first and all longitudinal survey rounds.

| Survey round and statistic | Variable |  |  |  |  |  |  |  |  |  |
| --- | --- | --- | --- | --- | --- | --- | --- | --- | --- | --- |
|  | 18-29 years | 30-39 years | 40-49 years | 50-59 years | 60-69 years | 70-79 years | Children at home | In a relationship | Female | N |
| **Round 2** |  |  |  |  |  |  |  |  |  |  |
| Survey round mean | 0.109** | 0.87* | 0.128* | 0.220 | 0.245** | 0.210*** | 0.267*** | 0.700 | 0.552 | 1,549 |
| Survey round std.dev. | 0.312 | 0.282 | 0.335 | 0.414 | 0.430 | 0.407 | 0.443 | 0.458 | 0.497 |  |
| Round 1 mean | 0.136 | 0.110 | 0.158 | 0.224 | 0.207 | 0.164 | 0.325 | 0.700 | 0.551 | 2,821 |
| Round 1 std.dev. | 0.343 | 0.313 | 0.365 | 0.417 | 0.405 | 0.370 | 0.469 | 0.458 | 0.497 |  |
| **Round 4** |  |  |  |  |  |  |  |  |  |  |
| Survey round mean | 0.089 | 0.079 | 0.135 | 0.225 | 0.261** | 0.210 | 0.268 | 0.706 | 0.557 | 1,632 |
| Survey round std.dev. | 0.285 | 0.270 | 0.342 | 0.418 | 0.439 | 0.408 | 0.443 | 0.456 | 0.497 |  |
| Round 1 mean | 0.136 | 0.110 | 0.158 | 0.224 | 0.207 | 0.164 | 0.325 | 0.700 | 0.551 | 2,821 |
| Round 1 std.dev. | 0.343 | 0.313 | 0.365 | 0.417 | 0.405 | 0.370 | 0.469 | 0.458 | 0.497 |  |
| **Round 6** |  |  |  |  |  |  |  |  |  |  |
| Survey round mean | 0.091 | 0.080 | 0.127 | 0.213 | 0.252** | 0.237 | 0.282 | 0.707 | 0.567 | 1,541 |
| Survey round std.dev. | 0.288 | 0.271 | 0.333 | 0.410 | 0.434 | 0.425 | 0.450 | 0.455 | 0.496 |  |
| Round 1 mean | 0.136 | 0.110 | 0.158 | 0.224 | 0.207 | 0.164 | 0.325 | 0.700 | 0.551 | 2,821 |
| Round 1 std.dev. | 0.343 | 0.313 | 0.365 | 0.417 | 0.405 | 0.370 | 0.469 | 0.458 | 0.497 |  |
| **Round 8** |  |  |  |  |  |  |  |  |  |  |
| Survey round mean | 0.073*** | 0.083 | 0.132 | 0.221 | 0.254*** | 0.237* | 0.289 | 0.703 | 0.555 | 1,513 |
| Survey round std.dev. | 0.260 | 0.276 | 0.339 | 0.415 | 0.436 | 0.425 | 0.454 | 0.457 | 0.497 |  |
| Round 1 mean | 0.136 | 0.110 | 0.158 | 0.224 | 0.207 | 0.164 | 0.325 | 0.700 | 0.551 | 2,821 |
| Round 1 std.dev. | 0.343 | 0.313 | 0.365 | 0.417 | 0.405 | 0.370 | 0.469 | 0.458 | 0.497 |  |
| **Round 10** |  |  |  |  |  |  |  |  |  |  |
| Survey round mean | 0.068** | 0.065 | 0.104 | 0.209 | 0.270*** | 0.284** | 0.245 | 0.707 | 0.553 | 938 |
| Survey round std.dev. | 0.252 | 0.247 | 0.306 | 0.407 | 0.444 | 0.451 | 0.430 | 0.455 | 0.497 |  |
| Round 1 mean | 0.136 | 0.110 | 0.158 | 0.224 | 0.207 | 0.164 | 0.325 | 0.700 | 0.551 | 2,821 |
| Round 1 std.dev. | 0.343 | 0.313 | 0.365 | 0.417 | 0.405 | 0.370 | 0.469 | 0.458 | 0.497 |  |
| *Notes:* Significance stars refer to tests of survey mean and standard deviation (std.dev.) against the mean and standard deviation (std.dev.) in Round 1. | | | | | | | | | | |
| * p < 0.05; ** p < 0.01; *** p < 0.001 (two-tailed tests). | | | | | | | | | | |

Table A3. Comparisons of means and standard deviations on key variables in first survey round, by pre/post lockdown responses.

| Survey round and statistic | Variable |  |  |  |  |  |  |  |  |  |
| --- | --- | --- | --- | --- | --- | --- | --- | --- | --- | --- |
|  | 18-29 years | 30-39 years | 40-49 years | 50-59 years | 60-69 years | 70-79 years | Children at home | In a relationship | Female | N |
| Pre-pandemic mean | 0.135 | 0.090 | 0.137 | 0.205 | 0.240 | 0.195 | 0.270 | 0.692 | 0.558 | 1,149 |
| Pre-pandemic std.dev. | 0.342 | 0.287 | 0.344 | 0.404 | 0.426 | 0.396 | 0.442 | 0.462 | 0.497 |  |
| Post pandemic mean | 0.138 | 0.120** | 0.173 | 0.237 | 0.190*** | 0.142 | 0.370*** | 0.706 | 0.547 | 1,672 |
| Post pandemic std.dev. | 0.345 | 0.329 | 0.379 | 0.426 | 0.389 | 0.350 | 0.482 | 0.456 | 0.498 |  |

*Notes:* Significance stars refer to tests of survey mean and standard deviation against the population mean and standard deviation within rounds.
* p < 0.05; ** p < 0.01; *** p < 0.001 (two-tailed tests).

Table A4: Point estimates and 95 % confidence intervals from regressions using 18-29-years-old within survey round as baseline.

|  | Survey round | | | | | | | | | | |
| --- | --- | --- | --- | --- | --- | --- | --- | --- | --- | --- | --- |
| Age group | Longitudinal  Mar 2-Mar 11, 2020 º | Longitudinal  Mar 12-Apr 13, 2020 | Longitudinal  Jul 2 - Aug 01, 2020 | Cross-sectional  Sept 01-Sept 30, 2020 | Longitudinal  Nov 04-Nov 30,2020 | Cross-sectional  Mar 03-Apr 24, 2021 | Longitudinal  Mar03-Apr 24, 2021 | Cross-sectional  Sept 14-Sept 26, 2021 | Longitudinal  Nov 12-Dec 30, 2021 | Cross-sectional  Mar 01-Apr 18, 2022 | Longitudinal  Mar 01-Apr 18, 2022 |
| 30-39 | -0.027 | 0.030 | -0.010 | -0.033 | -0.021 | -0.038 | -0.047 | -0.083^*^ | -0.023 | -0.115^**^ | 0.059 |
|  | (-0.15 : 0.10) | (-0.06 : 0.12) | (-0.11 : 0.09) | (-0.10 : 0.04) | (-0.13 : 0.09) | (-0.11-0.04) | (-0.16 : 0.07) | (-0.15 :  -0.02) | (-0.15 : 0.10) | (-0.19 :  -0.04) | (-0.10 : 0.22) |
| 40-49 | 0.069 | -0.014 | 0.002 | -0.024 | -0.127^*^ | -0.081^*^ | -0.057 | -0.147^***^ | -0.089 | -0.145^***^ | -0.006 |
|  | (-0.05 : 0.18) | (-0.09 : 0.06) | (-0.09 : 0.10) | (-0.09 : 0.05) | (-0.22 :  -0.03) | (-0.15 :  -0.01) | (-0.17 : 0.05) | (-0.21 :  -0.09) | (-0.20 : 0.03) | (-0.21 :  -0.08) | (-0.15 : 0.14) |
| 50-59 | -0.102^*^ | 0.008 | -0.035 | -0.034 | -0.123^**^ | -0.144^***^ | -0.171^***^ | -0.156^***^ | -0.166^**^ | -0.161^***^ | -0.006 |
|  | (-0.20 :  -0.00) | (-0.07 : 0.08) | (-0.12 : 0.05) | (-0.10 : 0.03) | (-0.21 :  -0.03) | (-0.21 :  -0.08) | (-0.27 :  -0.08) | (-0.21 :  -0.10) | (-0.27 :  -0.06) | (-0.22 :  -0.10) | (-0.13 : 0.12) |
| 60-69 | -0.137^**^ | -0.102^**^ | -0.085^*^ | -0.133^***^ | -0.136^**^ | -0.205^***^ | -0.109^*^ | -0.266^***^ | -0.191^***^ | -0.214^***^ | -0.009 |
|  | (-0.23 :  -0.04) | (-0.17 :  -0.03) | (-0.16 :  -0.01) | (-0.20 :  -0.07) | (-0.23 :  -0.05) | (-0.27 :  -0.15) | (-0.21 :  -0.01) | (-0.32 :  -0.21) | (-0.29 :  -0.09) | (-0.28 :  -0.15) | (-0.14 : 0.12) |
| 70-79 | -0.261^***^ | -0.111^**^ | -0.114^**^ | -0.149^***^ | -0.210^***^ | -0.266^***^ | -0.245^***^ | -0.300^***^ | -0.235^***^ | -0.307^***^ | -0.113 |
|  | (-0.35 :  -0.18) | (-0.19 :  -0.04) | (-0.19 :  -0.04) | (-0.21 :  -0.08) | (-0.30 :  -0.12) | (-0.32 : -0.21) | (-0.34 :  -0.15) | (-0.35 :  -0.25) | (-0.34 :  -0.13) | (-0.37 :  -0.25) | (-0.24 : 0.01) |
| Constant | 0.167 | 0.294^***^ | 0.201 | 0.258^*^ | 0.420^***^ | 0.463^***^ | 0.404^***^ | 0.451^***^ | 0.319^***^ | 0.404^***^ | 0.225^**^ |
| (18-29) | (-0.09 : 0.42) | (0.14 : 0.45) | (-0.05 : 0.45) | (0.05 : 0.47) | (0.22 :  0.61) | (0.41 : 0.52) | (0.29 : 0.52) | (0.32 : 0.58) | (0.21 : 0.43) | (0.33 : 0.48) | (0.07 : 0.38) |

*Notes:* Table reports parameter estimates and 95% confidence intervals (in parentheses) from regressing Pr(WHO5<50) on survey round by age fixed effects. All models control for weather as described under ‘Statistical Procedures’. Observations: 22,395. Population weights from Statistics Denmark were applied. º denotes responses prior to the first lockdown.
^***^ p<0.01- ^**^ p<0.05- ^*^ p<0.1 (two-tailed tests).

Table A5: Estimates and 95 % confidence intervals of the risk of stress/depression (Pr(WHO5<50)) by age groups and survey rounds.

|  | Survey round | | | | | | | | | | |
| --- | --- | --- | --- | --- | --- | --- | --- | --- | --- | --- | --- |
| Age group | Longitudinal  Mar 2-Mar 11, 2020 º | Longitudinal  Mar 12-Apr 13, 2020 | Longitudinal  Jul 2 - Aug 01, 2020 | Cross-sectional  Sept 01-Sept 30, 2020 | Longitudinal  Nov 04-Nov 30,2020 | Cross-sectional  Mar 03-Apr 24, 2021 | Longitudinal  Mar03-Apr 24, 2021 | Cross-sectional  Sept 14-Sept 26, 2021 | Longitudinal  Nov 12-Dec 30, 2021 | Cross-sectional  Mar 01-Apr 18, 2022 | Longitudinal  Mar 01-Apr 18, 2022 |
| 18-29 | 0.339^***^ | 0.255^***^ | 0.205^***^ | 0.293^***^ | 0.325^***^ | 0.428^***^ | 0.434^***^ | 0.428^***^ | 0.390^***^ | 0.478^***^ | 0.280^***^ |
|  | (0.259 : 0.419) | (0.195 : 0.316) | (0.130 : 0.280) | (0.233 : 0.352) | (0.244 : 0.406) | (0.377 : 0.478) | (0.346 : 0.521) | (0.375 : 0.481) | (0.292 : 0.488) | (0.423 : 0.532) | (0.167 : 0.393) |
| 30-39 | 0.311^***^ | 0.296^***^ | 0.210^***^ | 0.261^***^ | 0.273^***^ | 0.397^***^ | 0.358^***^ | 0.348^***^ | 0.347^***^ | 0.362^***^ | 0.426^***^ |
|  | (0.214 : 0.408) | (0.228 : 0.363) | (0.129 : 0.291) | (0.203 : 0.319) | (0.190 : 0.355) | (0.339 : 0.455) | (0.267 : 0.448) | (0.291 : 0.405) | (0.257 : 0.437) | (0.302 : 0.421) | (0.297 : 0.555) |
| 40-49 | 0.398^***^ | 0.244^***^ | 0.195^***^ | 0.269^***^ | 0.191^***^ | 0.348^***^ | 0.361^***^ | 0.279^***^ | 0.280^***^ | 0.333^***^ | 0.262^***^ |
|  | (0.316 : 0.481) | (0.190 : 0.297) | (0.125 : 0.265) | (0.212 : 0.326) | (0.131 : 0.252) | (0.298 : 0.398) | (0.282 : 0.440) | (0.229 : 0.330) | (0.208 : 0.351) | (0.283 : 0.383) | (0.164 : 0.361) |
| 50-59 | 0.234^***^ | 0.268^***^ | 0.170^***^ | 0.258^***^ | 0.190^***^ | 0.291^***^ | 0.253^***^ | 0.272^***^ | 0.259^***^ | 0.315^***^ | 0.288^***^ |
|  | (0.174 : 0.294) | (0.217 : 0.319) | (0.113 : 0.227) | (0.207 : 0.309) | (0.143 : 0.238) | (0.249 : 0.333) | (0.198 : 0.308) | (0.227 : 0.317) | (0.204 : 0.314) | (0.271 : 0.359) | (0.212 : 0.364) |
| 60-69 | 0.203^***^ | 0.159^***^ | 0.127^***^ | 0.161^***^ | 0.171^***^ | 0.225^***^ | 0.306^***^ | 0.164^***^ | 0.185^***^ | 0.264^***^ | 0.282^***^ |
|  | (0.149 : 0.258) | (0.115 : 0.204) | (0.075 : 0.179) | (0.115 : 0.207) | (0.125 : 0.217) | (0.187 : 0.262) | (0.247 : 0.364) | (0.121 : 0.206) | (0.138 : 0.232) | (0.222 : 0.306) | (0.209 : 0.355) |
| 70-79 | 0.078^***^ | 0.148^***^ | 0.095^***^ | 0.145^***^ | 0.110^***^ | 0.168^***^ | 0.172^***^ | 0.126^***^ | 0.158^***^ | 0.169^***^ | 0.215^***^ |
|  | (0.041 : 0.116) | (0.098 : 0.198) | (0.046 : 0.144) | (0.097 : 0.193) | (0.068 : 0.151) | (0.133 : 0.204) | (0.124 : 0.220) | (0.083 : 0.169) | (0.113 : 0.204) | (0.132 : 0.206) | (0.151 : 0.279) |

*Notes:* Table reports parameter estimates and 95% confidence intervals (in parentheses) from regressing Pr(WHO5<50) on survey round by age fixed effects. All models control for weather as described under ‘Statistical Procedures’. Observations: 22,395. Population weights from Statistics Denmark were applied. º denotes responses prior to the first lockdown.
^***^ p<0.01, ^**^ p<0.05, ^*^ p<0.1 (two-tailed tests).

Table A6: Estimates and 95 % confidence intervals of the risk of stress/depression (Pr(WHO5<50)) by gender, living arrangements and survey rounds.

|  | Survey round | | | | | | | | | | |
| --- | --- | --- | --- | --- | --- | --- | --- | --- | --- | --- | --- |
|  | Longitudinal (March 2-March 11, 2020) º | Longitudinal (March 12-April 13, 2020) | Longitudinal (July 2 - August 01, 2020) | Cross-sectional (September 01-September 30, 2020) | Longitudinal (November 04-November 30,2020) | Cross-sectional (March 03-April 24, 2021) | Longitudinal (March 03-April 24, 2021) | Cross-sectional (September 14-September 26, 2021) | Longitudinal (November 12-December 30, 2021) | Cross-sectional (March 01-April 18, 2022) | Longitudinal (March 01-April 18, 2022) |
| **Male** |  |  |  |  |  |  |  |  |  |  |  |
| No children | 0.240*** | 0.247*** | 0.167*** | 0.231*** | 0.193*** | 0.274*** | 0.290*** | 0.221*** | 0.212*** | 0.292*** | 0.254*** |
|  | (0.187 : 0.292) | (0.200 : 0.294) | (0.118 : 0.217) | (0.186 : 0.275) | (0.149 : 0.238) | (0.240 : 0.308) | (0.237 : 0.344) | (0.180 : 0.261) | (0.164 : 0.260) | (0.254 : 0.331) | (0.187 : 0.320) |
| Children | 0.315*** | 0.181*** | 0.128*** | 0.218*** | 0.118*** | 0.270*** | 0.211*** | 0.254*** | 0.214*** | 0.309*** | 0.259*** |
|  | (0.218 : 0.413) | (0.131 : 0.231) | (0.060 : 0.195) | (0.160 : 0.275) | (0.060 : 0.177) | (0.221 : 0.319) | (0.137 : 0.284) | (0.202 : 0.306) | (0.140 : 0.287) | (0.255 : 0.363) | (0.157 : 0.361) |
| **Female** |  |  |  |  |  |  |  |  |  |  |  |
| No children | 0.227*** | 0.234*** | 0.190*** | 0.231*** | 0.275*** | 0.330*** | 0.358*** | 0.322*** | 0.337*** | 0.341*** | 0.345*** |
|  | (0.181 : 0.274) | (0.192 : 0.276) | (0.139 : 0.240) | (0.187 : 0.275) | (0.226 : 0.324) | (0.295 : 0.364) | (0.302 : 0.414) | (0.281 : 0.364) | (0.284 : 0.389) | (0.303 : 0.380) | (0.275 : 0.415) |
| Children | 0.330*** | 0.268*** | 0.178*** | 0.297*** | 0.246*** | 0.377*** | 0.359*** | 0.369*** | 0.301*** | 0.358*** | 0.257*** |
|  | (0.259 : 0.402) | (0.215 : 0.321) | (0.115 : 0.240) | (0.246 : 0.349) | (0.185 : 0.307) | (0.330 : 0.425) | (0.291 : 0.428) | (0.320 : 0.418) | (0.235 : 0.367) | (0.310 : 0.406) | (0.169 : 0.344) |
| *Notes:* Table reports parameter estimates and 95% confidence intervals (in parentheses) from regressing Pr(WHO5<50) on survey round by gender and living arrangements fixed effects. All models control for weather as described under ‘Statistical Procedures’. Observations: 22,395. Population weights from Statistics Denmark were applied. º denotes responses prior to the first lockdown. ^***^ p<0.01, ^**^ p<0.05, ^*^ p<0.1 (two-tailed tests). | | | | | | | | | | | |

Table A7: Point estimates and 95 % confidence intervals from regressions using men without children at home as baseline within survey round.

|  | Survey round | | | | | | | | | | |
| --- | --- | --- | --- | --- | --- | --- | --- | --- | --- | --- | --- |
|  | Longitudinal (March 2-March 11- 2020) º | Longitudinal (March 12-April 13- 2020) | Longitudinal (July 2 - August 01- 2020) | Cross-sectional (September 01-September 30- 2020) | Longitudinal (November 04-November 30-2020) | Cross-sectional (March 03-April 24- 2021) | Longitudinal (March 03-April 24- 2021) | Cross-sectional (September 14-September 26- 2021) | Longitudinal (November 12-December 30- 2021) | Cross-sectional (March 01-April 18- 2022) | Longitudinal (March 01-April 18- 2022) |
| **Male** |  |  |  |  |  |  |  |  |  |  |  |
| Constant | 0.116 | 0.270^***^ | 0.152 | 0.202 | 0.314^**^ | 0.301^***^ | 0.268^***^ | 0.232^***^ | 0.188^***^ | 0.220^***^ | 0.179^**^ |
| (without children) | (-0.14 : 0.37) | (0.12 : 0.42) | (-0.09 : 0.40) | (-0.01 : 0.42) | (0.12 : 0.51) | (0.26 : 0.34) | (0.18 : 0.36) | (0.10 : 0.36) | (0.10 : 0.28) | (0.16 : 0.29) | (0.05 : 0.31) |
| Children | 0.077 | -0.066 | -0.041 | -0.014 | -0.074^*^ | -0.004 | -0.081 | 0.033 | 0.004 | 0.017 | -0.004 |
|  | (-0.03 : 0.19) | (-0.13 : 0.00) | (-0.11 : 0.03) | (-0.07 : 0.05) | (-0.14 :  -0.00) | (-0.06 : 0.05) | (-0.17 : 0.00) | (-0.02 : 0.09) | (-0.08 : 0.09) | (-0.04 : 0.08) | (-0.12 : 0.11) |
| **Female** |  |  |  |  |  |  |  |  |  |  |  |
| No children | -0.012 | -0.013 | 0.022 | -0.000 | 0.083^*^ | 0.054^*^ | 0.068 | 0.101^***^ | 0.125^***^ | 0.048^*^ | 0.086 |
|  | (-0.08 : 0.06) | (-0.07 : 0.05) | (-0.03 : 0.08) | (-0.05 : 0.05) | (0.02 : 0.15) | (0.01 : 0.10) | (-0.00 : 0.14) | (0.06 : 0.14) | (0.06 : 0.19) | (0.00 : 0.09) | (-0.00 : 0.18) |
| Children | 0.093^*^ | 0.022 | 0.010 | 0.066^*^ | 0.054 | 0.102^***^ | 0.069 | 0.147^***^ | 0.092^*^ | 0.063^*^ | -0.008 |
|  | (0.01 : 0.18) | (-0.05 : 0.09) | (-0.06 : 0.08) | (0.01 : 0.12) | (-0.02 : 0.13) | (0.05 : 0.16) | (-0.01 : 0.15) | (0.10 : 0.20) | (0.01 : 0.17) | (0.01 : 0.12) | (-0.11 : 0.10) |
| *Notes:* Table reports parameter estimates and 95% confidence intervals (in parentheses) from regressing Pr(WHO5<50) on survey round by gender and living arrangements fixed effects. All models control for weather as described under ‘Statistical Procedures’. Observations: 22,395. Population weights from Statistics Denmark were applied. º denotes responses prior to the first lockdown. ^***^ p<0.01- ^**^ p<0.05- ^*^ p<0.1 (two-tailed tests). | | | | | | | | | | | |

Table A8: Estimates and 95 % confidence intervals of WHO5 index (full-scale) by age groups and survey rounds.

|  | Survey round | | | | | | | | | | |
| --- | --- | --- | --- | --- | --- | --- | --- | --- | --- | --- | --- |
| Age group | Longitudinal (March 2-March 11, 2020) º | Longitudinal (March 12-April 13, 2020) | Longitudinal (July 2 - August 01, 2020) | Cross-sectional (September 01-September 30, 2020) | Longitudinal (November 04-November 30,2020) | Cross-sectional (March 03-April 24, 2021) | Longitudinal (March 03-April 24, 2021) | Cross-sectional (September 14-September 26, 2021) | Longitudinal (November 12-December 30, 2021) | Cross-sectional (March 01-April 18, 2022) | Longitudinal (March 01-April 18, 2022) |
| 18-29 | 56.353*** | 61.398*** | 63.845*** | 59.245*** | 57.657*** | 53.116*** | 52.431*** | 52.956*** | 55.744*** | 51.220*** | 61.538*** |
|  | (52.392 : 60.314) | (58.427 : 64.369) | (60.718 : 66.972) | (56.640 : 61.851) | (54.151 : 61.163) | (50.906 : 55.326) | (48.836 : 56.027) | (50.575 : 55.337) | (51.811 : 59.677) | (48.768 : 53.673) | (56.511 : 66.565) |
| 30-39 | 59.777*** | 60.741*** | 63.113*** | 60.854*** | 59.579*** | 55.483*** | 56.459*** | 56.373*** | 56.037*** | 56.782*** | 53.110*** |
|  | (55.132 : 64.423) | (57.453 : 64.029) | (59.279 : 66.947) | (58.200 : 63.508) | (56.083 : 63.075) | (52.849 : 58.117) | (52.380 : 60.537) | (53.779 : 58.968) | (52.183 : 59.891) | (53.883 : 59.680) | (47.665 : 58.554) |
| 40-49 | 55.967*** | 62.385*** | 64.624*** | 61.397*** | 62.569*** | 58.610*** | 56.806*** | 60.492*** | 58.599*** | 57.889*** | 59.187*** |
|  | (51.953 : 59.980) | (59.645 : 65.125) | (61.217 : 68.031) | (58.896 : 63.899) | (59.740 : 65.398) | (56.247 : 60.973) | (53.047 : 60.566) | (58.130 : 62.854) | (55.453 : 61.745) | (55.460 : 60.319) | (54.353 : 64.022) |
| 50-59 | 62.994*** | 62.883*** | 67.292*** | 63.222*** | 65.295*** | 60.789*** | 62.246*** | 61.553*** | 62.098*** | 60.074*** | 60.868*** |
|  | (59.791 : 66.197) | (60.220 : 65.546) | (64.471 : 70.113) | (60.844 : 65.600) | (63.178 : 67.413) | (58.705 : 62.873) | (59.709 : 64.783) | (59.298 : 63.809) | (59.619 : 64.578) | (57.906 : 62.241) | (57.123 : 64.613) |
| 60-69 | 67.253*** | 69.735*** | 70.497*** | 68.882*** | 66.318*** | 65.841*** | 61.573*** | 69.320*** | 65.617*** | 65.718*** | 62.486*** |
|  | (64.187 : 70.320) | (67.254 : 72.215) | (67.897 : 73.097) | (66.715 : 71.049) | (63.910 : 68.726) | (63.753 : 67.930) | (58.621 : 64.525) | (67.176 : 71.465) | (63.163 : 68.070) | (63.634 : 67.801) | (58.905 : 66.067) |
| 70-79 | 75.331*** | 71.265*** | 72.927*** | 69.629*** | 71.262*** | 69.352*** | 66.804*** | 71.180*** | 68.625*** | 70.565*** | 65.130*** |
|  | (72.984 : 77.678) | (68.442 : 74.088) | (70.464 : 75.390) | (67.183 : 72.076) | (68.839 : 73.685) | (67.361 : 71.344) | (64.297 : 69.311) | (68.946 : 73.413) | (66.188 : 71.062) | (68.557 : 72.572) | (61.699 : 68.562) |
| *Notes:* Table reports parameter estimates and 95% confidence intervals (in parentheses) from regressing WHO5 on survey round by age fixed effects. All models control for weather as described under ‘Statistical Procedures’. Observations: 22,395. Population weights from Statistics Denmark were applied. º denotes responses prior to the first lockdown. ^***^ p<0.01, ^**^ p<0.05, ^*^ p<0.1 (two-tailed tests). | | | | | | | | | | | |

Table A9: Estimates and 95 % confidence intervals of WHO5 index (full-scale) by gender, living arrangements and survey rounds.

|  | Survey round | | | | | | | | | | |
| --- | --- | --- | --- | --- | --- | --- | --- | --- | --- | --- | --- |
|  | Longitudinal (March 2-March 11, 2020) º | Longitudinal (March 12-April 13, 2020) | Longitudinal (July 2 - August 01, 2020) | Cross-sectional (September 01-September 30, 2020) | Longitudinal (November 04-November 30,2020) | Cross-sectional (March 03-April 24, 2021) | Longitudinal (March 03-April 24, 2021) | Cross-sectional (September 14-September 26, 2021) | Longitudinal (November 12-December 30, 2021) | Cross-sectional (March 01-April 18, 2022) | Longitudinal (March 01-April 18, 2022) |
| **Male** |  |  |  |  |  |  |  |  |  |  |  |
| No children | 65.093*** | 64.397*** | 67.630*** | 64.432*** | 65.550*** | 63.078*** | 61.352*** | 64.504*** | 64.276*** | 63.225*** | 62.651*** |
|  | (62.350 : 67.837) | (62.002 : 66.792) | (65.204 : 70.056) | (62.383 : 66.480) | (63.488 : 67.613) | (61.340 : 64.816) | (58.688 : 64.017) | (62.546 : 66.462) | (61.948 : 66.603) | (61.360 : 65.090) | (59.412 : 65.889) |
| Children | 57.798*** | 66.083*** | 68.793*** | 63.242*** | 66.876*** | 61.455*** | 64.030*** | 61.694*** | 63.713*** | 60.366*** | 62.348*** |
|  | (52.937 : 62.659) | (63.428 : 68.737) | (65.854 : 71.732) | (60.645 : 65.840) | (63.835 : 69.916) | (59.273 : 63.636) | (60.696 : 67.364) | (59.229 : 64.160) | (60.521 : 66.906) | (57.664 : 63.068) | (57.018 : 67.677) |
| **Female** |  |  |  |  |  |  |  |  |  |  |  |
| No children | 64.554*** | 64.705*** | 66.112*** | 63.960*** | 60.727*** | 59.106*** | 57.197*** | 59.997*** | 58.543*** | 58.871*** | 58.499*** |
|  | (62.157 : 66.950) | (62.623 : 66.786) | (63.796 : 68.428) | (61.923 : 65.997) | (58.565 : 62.889) | (57.414 : 60.798) | (54.743 : 59.652) | (57.993 : 62.000) | (56.270 : 60.816) | (57.014 : 60.729) | (55.425 : 61.574) |
| Children | 58.311*** | 61.137*** | 65.777*** | 60.329*** | 60.918*** | 56.837*** | 56.273*** | 56.387*** | 57.868*** | 56.806*** | 60.395*** |
|  | (54.690 : 61.932) | (58.439 : 63.836) | (62.781 : 68.774) | (58.048 : 62.610) | (58.203 : 63.633) | (54.663 : 59.011) | (53.315 : 59.231) | (54.156 : 58.618) | (55.042 : 60.694) | (54.591 : 59.021) | (56.597 : 64.193) |
| *Notes:* Table reports parameter estimates and 95% confidence intervals (in parentheses) from regressing WHO5 on survey round by gender and living arrangements fixed effects. All models control for weather as described under ‘Statistical Procedures’. Observations: 22,395. Population weights from Statistics Denmark were applied. º denotes responses prior to the first lockdown. ^***^ p<0.01, ^**^ p<0.05, ^*^ p<0.1 (two-tailed tests). | | | | | | | | | | | |

Table A10: Estimates and 95 % confidence intervals of the risk of stress/depression (Pr(WHO5<50)) by fixed age groups and survey rounds.

|  | Survey round | | | | | | | | | | |
| --- | --- | --- | --- | --- | --- | --- | --- | --- | --- | --- | --- |
| Age group | Longitudinal (March 2-March 11, 2020) º | Longitudinal (March 12-April 13, 2020) | Longitudinal (July 2 - August 01, 2020) | Cross-sectional (September 01-September 30, 2020) | Longitudinal (November 04-November 30,2020) | Cross-sectional (March 03-April 24, 2021) | Longitudinal (March 03-April 24, 2021) | Cross-sectional (September 14-September 26, 2021) | Longitudinal (November 12-December 30, 2021) | Cross-sectional (March 01-April 18, 2022) | Longitudinal (March 01-April 18, 2022) |
| 18-29 | 0.339*** | 0.260*** | 0.207*** | 0.293*** | 0.317*** | 0.429*** | 0.416*** | 0.428*** | 0.378*** | 0.477*** | 0.308*** |
|  | (0.259 : 0.419) | (0.199 : 0.320) | (0.132 : 0.282) | (0.234 : 0.352) | (0.237 : 0.397) | (0.379 : 0.480) | (0.333 : 0.500) | (0.375 : 0.481) | (0.287 : 0.470) | (0.423 : 0.532) | (0.198 : 0.418) |
| 30-39 | 0.311*** | 0.289*** | 0.196*** | 0.260*** | 0.283*** | 0.390*** | 0.369*** | 0.345*** | 0.351*** | 0.362*** | 0.370*** |
|  | (0.214 : 0.409) | (0.221 : 0.357) | (0.117 : 0.275) | (0.202 : 0.318) | (0.203 : 0.364) | (0.332 : 0.448) | (0.279 : 0.458) | (0.288 : 0.402) | (0.264 : 0.437) | (0.303 : 0.421) | (0.250 : 0.490) |
| 40-49 | 0.399*** | 0.245*** | 0.208*** | 0.269*** | 0.183*** | 0.350*** | 0.359*** | 0.281*** | 0.293*** | 0.333*** | 0.287*** |
|  | (0.316 : 0.481) | (0.191 : 0.299) | (0.137 : 0.279) | (0.212 : 0.325) | (0.125 : 0.242) | (0.300 : 0.400) | (0.279 : 0.439) | (0.230 : 0.332) | (0.221 : 0.366) | (0.283 : 0.384) | (0.186 : 0.388) |
| 50-59 | 0.234*** | 0.267*** | 0.171*** | 0.259*** | 0.186*** | 0.290*** | 0.245*** | 0.273*** | 0.215*** | 0.315*** | 0.291*** |
|  | (0.174 : 0.294) | (0.217 : 0.318) | (0.114 : 0.227) | (0.207 : 0.310) | (0.139 : 0.232) | (0.248 : 0.332) | (0.192 : 0.298) | (0.227 : 0.318) | (0.168 : 0.263) | (0.272 : 0.359) | (0.218 : 0.364) |
| 60-69 | 0.204*** | 0.158*** | 0.121*** | 0.161*** | 0.172*** | 0.228*** | 0.307*** | 0.162*** | 0.195*** | 0.262*** | 0.278*** |
|  | (0.149 : 0.258) | (0.113 : 0.203) | (0.069 : 0.174) | (0.114 : 0.207) | (0.126 : 0.219) | (0.190 : 0.266) | (0.249 : 0.366) | (0.119 : 0.205) | (0.145 : 0.245) | (0.220 : 0.305) | (0.205 : 0.351) |
| 70-79 | 0.078*** | 0.148*** | 0.092*** | 0.145*** | 0.103*** | 0.165*** | 0.172*** | 0.127*** | 0.150*** | 0.169*** | 0.187*** |
|  | (0.041 : 0.116) | (0.098 : 0.198) | (0.043 : 0.141) | (0.097 : 0.193) | (0.061 : 0.146) | (0.130 : 0.200) | (0.121 : 0.223) | (0.084 : 0.171) | (0.101 : 0.198) | (0.132 : 0.206) | (0.120 : 0.254) |
| *Notes:* Table reports parameter estimates and 95% confidence intervals (in parentheses) from regressing Pr(WHO5<50) on survey round by age groups fixed effects. All models control for weather as described under ‘Statistical Procedures’. Observations: 22,395. Population weights from Statistics Denmark were applied.  º denotes responses prior to the first lockdown. ^***^ p<0.01, ^**^ p<0.05, ^*^ p<0.1 (two-tailed tests). | | | | | | | | | | | |

Figure A1: Estimates and 95 % confidence intervals of the risk of stress/depression (Pr(WHO5<50)) by age groups and survey rounds.
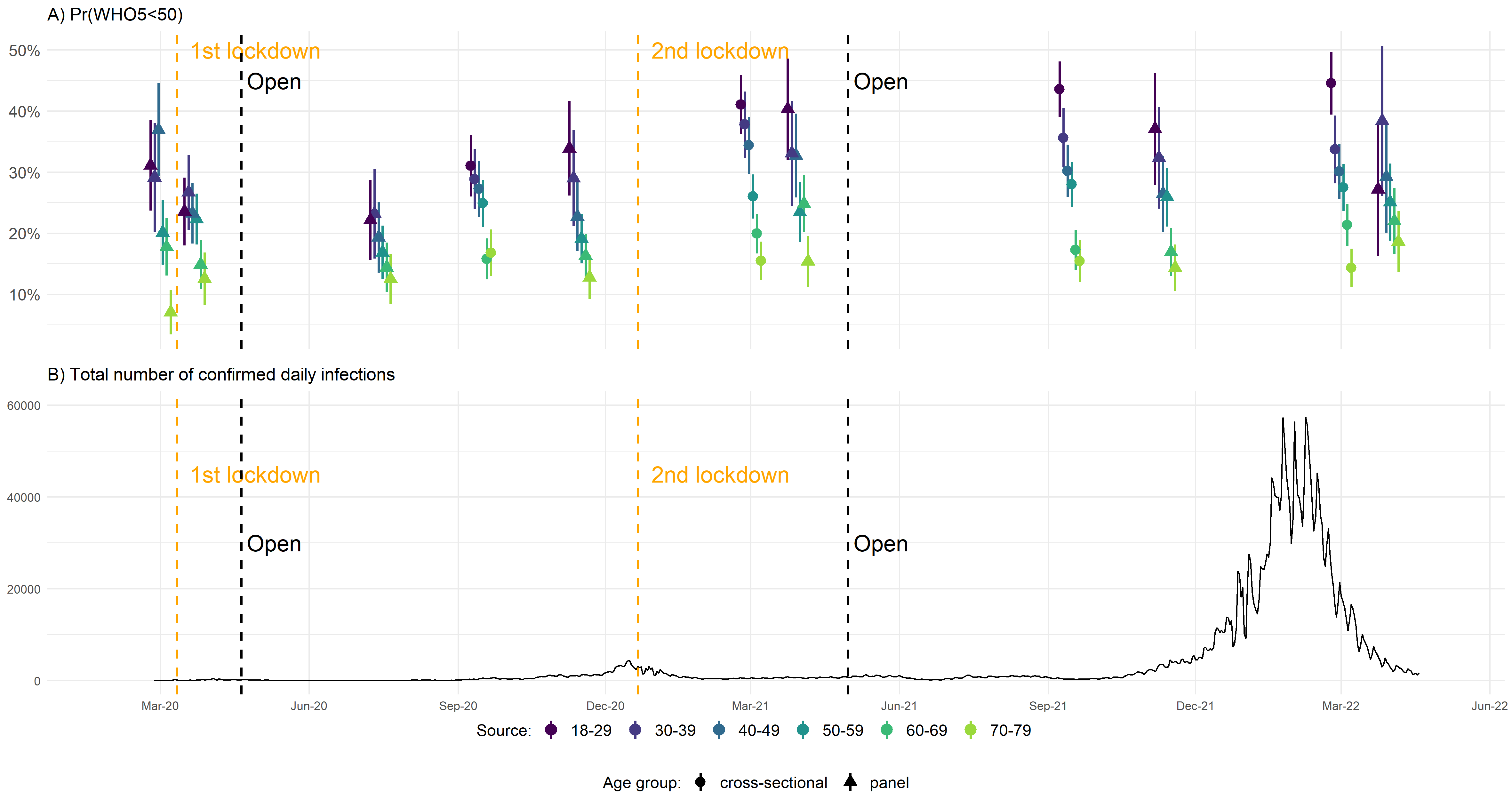


*Notes:* Panel A) reports parameter estimates and 95% confidence intervals from regressing Pr(WHO5<50) on survey round by age fixed effects. All models control for weather as described under ‘Statistical Procedures’. Observations: 22,395. No population weights were applied. Panel B) reports the total number of confirmed daily infection in Denmark.

Figure A2: Estimates and 95 % confidence intervals of the risk of stress/depression (Pr(WHO5<50)) by gender, living arrangements and survey rounds.


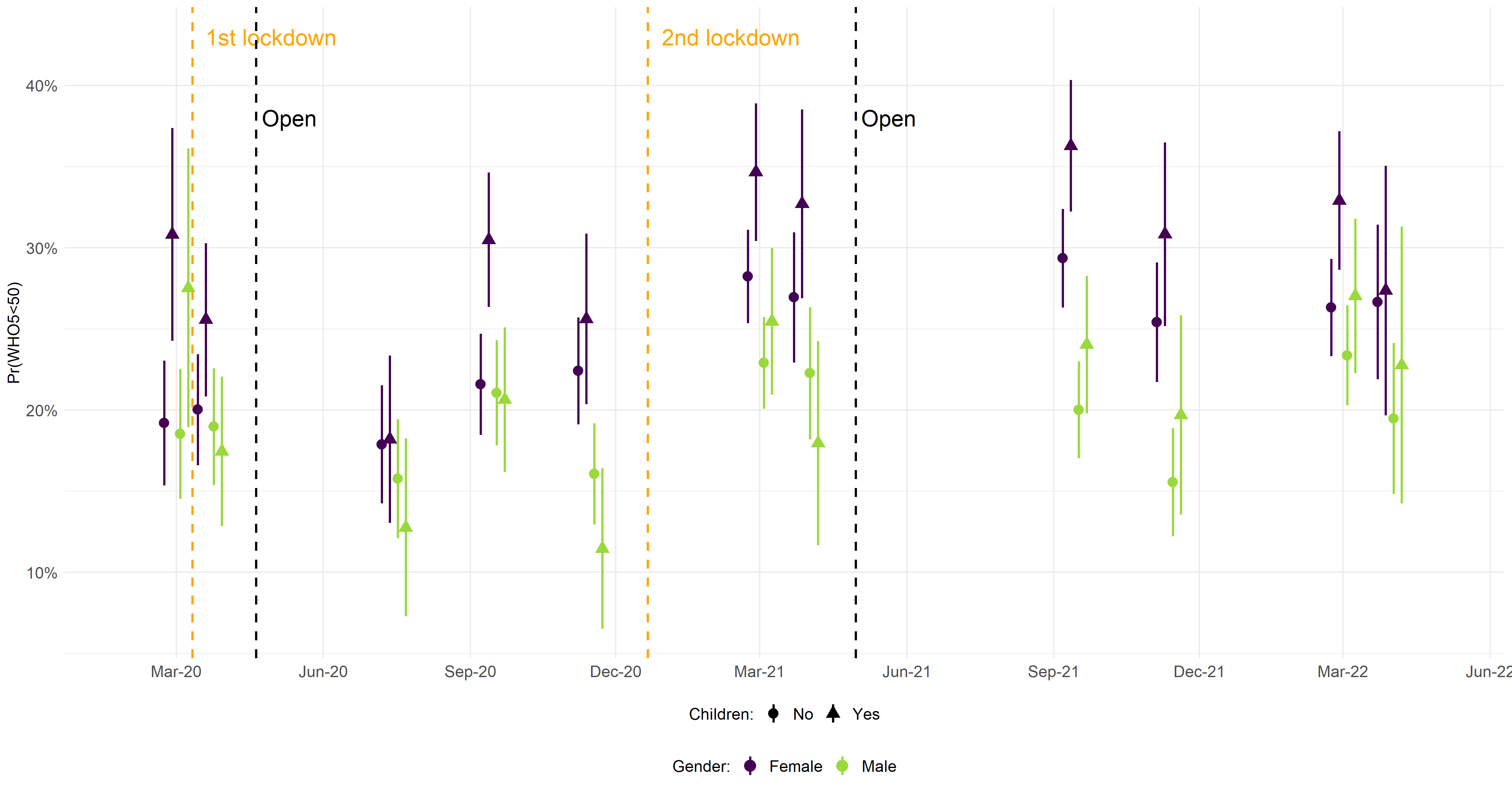


*Notes:* Figure A2 reports parameter estimates and 95% confidence intervals from regressing Pr(WHO5<50) on survey round by gender and living arrangements fixed effects. All models control for weather as described under ‘Statistical Procedures’. Observations: 22,395. No population weights were applied.

**Appendix B**

Changes in the local threat of infection could be important for wellbeing, which is why we here analyze the association between local infection rates and the risk of stress/depression. We measure the local infection rate as the number of confirmed positive cases per 100,000 inhabitants in the municipality in which the respondent lives (publicly available official daily test data from Statens Serum Institut). To overcome idiosyncratic variation in local infection rates we in practice use a 14-days moving average of the local infection rate, and to handle right-skewness in local infection rates we use log-transformation. The Danish testing strategy evolved drastically across the pandemic^1^, so we compared infection rates within survey waves only, utilizing that the variation between municipalities in infection rates was large.^2^ Denmark built up extensive testing capacity, with over 65 million PCR and 61 million antigen tests conducted up to May 9, 2022.

We used a binned scatter analysis of the functional form of the relationship between being at risk of stress/depression and the local infection rate. The binned scatter analysis combines a non-parametric estimate of the Conditional Expectation Function (CEF) and the best linear estimate of the CEF. The combination allows us to observe whether changes in local infection rates relate to changes in the outcome, Pr(WHO5 < 50), across our data period. We run two specifications (one for age groups and one for gender and living arrangements) similarly to our main specifications. And we reproduce the results for the full WHO5 scale for robustness.

Tables B1 and B2 report the results from the binned scatter analysis with Pr(WHO5 < 50) as the outcome variables, and Tables B3 and B4 report the results from the robustness analysis of the full WHO5 scale. We observe no consistently significant associations between local infection rates and the risk of being at risk of stress/depression (nor using the full scale). In conclusion, we rule out important confounding of local infection rates and the risk of stress/depression, implying that our main results are likely robust to changes in local infection rates.

*References*

1 Busk PK, Kristiansen TB, Engsig-Karup A. Impact of the national test strategy on the development of the Covid-19 pandemic in Denmark. *medRxiv* 2021; : 2021.07.08.21260182.

2 Holmager TL, Lynge E, Kann CE, St-Martin G. Geography of COVID-19 in Denmark. *Scand J Public Health* 2021; **49**: 88–95.

Table B1: Estimates and standard errors of local infections rates on the risk of stress/depression (Pr(WHO5 < 50)) by age groups and survey rounds.

|  | Survey rounds | | | | | | | | | | | | | | | |
| --- | --- | --- | --- | --- | --- | --- | --- | --- | --- | --- | --- | --- | --- | --- | --- | --- |
|  | Longitudinal (March 2-March 11, 2020) º | Longitudinal (March 12-April 13, 2020) | Longitudinal (July 2 - August 01, 2020) | Cross-sectional (September 01-September 30, 2020) | Longitudinal (November 04-November 30,2020) | Cross-sectional (March 03-April 24, 2021) | | Longitudinal (March 03-April 24, 2021) | | Cross-sectional (September 14-September 26, 2021) | | Longitudinal (November 12-December 30, 2021) | | Cross-sectional (March 01-April 18, 2022) | | Longitudinal (March 01-April 18, 2022) |
| *log(*local infection rate*)* | -0.002 | -0.005 | 0.008 | -0.002 | -0.001 | -0.002 | -0.012 | | 0.000 | | -0.010 | | 0.007 | | 0.043 | |
|  | (0.023) | (0.013) | (0.012) | (0.009) | (0.020) | (0.012) | | (0.017) | | (0.010) | | (0.022) | | (0.019) | | (0.024) |
|  |  |  |  |  |  |  | |  | |  | |  | |  | |  |
| *N* | 310 | 1,617 | 1,241 | 2,727 | 1,589 | 2,974 | | 1,489 | | 3,667 | | 1,459 | | 3,182 | | 914 |
| *Notes:* Table reports parameter estimates and standard errors (in parentheses) from binned regressions of Pr(WHO5<50) on log(local infection rate) by survey round and age groups fixed effects. All models control for weather as described under ‘Statistical Procedures’. Population weights from Statistics Denmark were applied.  º denotes responses prior to the first lockdown. ^***^ p<0.01, ^**^ p<0.05, ^*^ p<0.1 (two-tailed tests). | | | | | | | | | | | | | | | | |

Table B2: Estimates and standard errors of local infections rates on the risk of stress/depression (Pr(WHO5 < 50)) by gender, living arrangements and survey rounds.

|  | Survey round | | | | | | | | | | |
| --- | --- | --- | --- | --- | --- | --- | --- | --- | --- | --- | --- |
|  | Longitudinal (March 2-March 11, 2020) º | Longitudinal (March 12-April 13, 2020) | Longitudinal (July 2 - August 01, 2020) | Cross-sectional (September 01-September 30, 2020) | Longitudinal (November 04-November 30,2020) | Cross-sectional (March 03-April 24, 2021) | Longitudinal (March 03-April 24, 2021) | Cross-sectional (September 14-September 26, 2021) | Longitudinal (November 12-December 30, 2021) | Cross-sectional (March 01-April 18, 2022) | Longitudinal (March 01-April 18, 2022) |
| *log(*local infection rate*)* | -0.017 | 0.003 | 0.012 | 0.005 | 0.013 | 0.011 | -0.001 | 0.016 | 0.002 | -0.005 | 0.034 |
|  | (0.023) | (0.013) | (0.012) | (0.007) | (0.020) | (0.013) | (0.017) | (0.010) | (0.022) | (0.019) | (0.024) |
|  |  |  |  |  |  |  |  |  |  |  |  |
| *N* | 310 | 1,617 | 1,241 | 2,727 | 1,589 | 2,974 | 1,489 | 3,667 | 1,459 | 3,182 | 914 |
| *Notes:* Table reports parameter estimates and standard errors (in parentheses) from binned regressions of Pr(WHO5<50) on log(local infection rate) by survey round, gender and living arrangements fixed effects. All models control for weather as described under ‘Statistical Procedures’. Population weights from Statistics Denmark were applied.  º denotes responses prior to the first lockdown. ^***^ p<0.01, ^**^ p<0.05, ^*^ p<0.1 (two-tailed tests). | | | | | | | | | | | |

Table B3: Estimates and standard errors of local infections rates on the WHO5 index (full-scale) by age groups and survey rounds.

|  | Survey round | | | | | | | | | | | |
| --- | --- | --- | --- | --- | --- | --- | --- | --- | --- | --- | --- | --- |
|  | Longitudinal (March 2-March 11, 2020) º | Longitudinal (March 12-April 13, 2020) | Longitudinal (July 2 - August 01, 2020) | Cross-sectional (September 01-September 30, 2020) | Longitudinal (November 04-November 30,2020) | Cross-sectional (March 03-April 24, 2021) | Longitudinal (March 03-April 24, 2021) | Cross-sectional (September 14-September 26, 2021) | Longitudinal (November 12-December 30, 2021) | Cross-sectional (March 01-April 18, 2022) | Longitudinal (March 01-April 18, 2022) |  |
| log(local infection rate) | 1.078 | 0.326 | -0.320 | -0.027 | -1.000 | -0.246 | -0.647 | -0.028 | 0.297 | -0.142 | -1.238 |  |
|  | (1.178) | (0.647) | (0.571) | (0.408) | (0.924) | (0.606) | (0.781) | (0.496) | (0.995) | (0.918) | (1.143) |  |
|  |  |  |  |  |  |  |  |  |  |  |  |  |
| N | 310 | 1617 | 1241 | 2727 | 1589 | 2974 | 1489 | 3667 | 1459 | 3182 | 914 |  |
| *Notes:* Table reports parameter estimates and standard errors (in parentheses) from binned regressions on WHO5 index on log(local infection rate) by survey round and age groups fixed effects. All models control for weather as described under ‘Statistical Procedures’. Population weights from Statistics Denmark were applied.  º denotes responses prior to the first lockdown. ^***^ p<0.01, ^**^ p<0.05, ^*^ p<0.1 (two-tailed tests). | | | | | | | | | | | |  |

Table B4: Estimates and standard errors of local infections rates on the WHO5 index (full-scale) by gender, living arrangements and survey rounds.

|  | Survey round | | | | | | | | | | |
| --- | --- | --- | --- | --- | --- | --- | --- | --- | --- | --- | --- |
|  | Longitudinal (March 2-March 11, 2020) º | Longitudinal (March 12-April 13, 2020) | Longitudinal (July 2 - August 01, 2020) | Cross-sectional (September 01-September 30, 2020) | Longitudinal (November 04-November 30,2020) | Cross-sectional (March 03-April 24, 2021) | Longitudinal (March 03-April 24, 2021) | Cross-sectional (September 14-September 26, 2021) | Longitudinal (November 12-December 30, 2021) | Cross-sectional (March 01-April 18, 2022) | Longitudinal (March 01-April 18, 2022) |
| *log(*local infection rate*)* | 2.193 | -0.328 | -0.627 | -0.575 | -1.993^*^ | -0.995 | -1.232 | -1.056^*^ | -0.349 | 0.789 | -0.416 |
|  | (1.192) | (0.647) | (0.577) | (0.409) | (0.923) | (0.62) | (0.786) | (0.509) | (1.008) | (0.944) | (1.147) |
|  |  |  |  |  |  |  |  |  |  |  |  |
| *N* | 310 | 1617 | 1241 | 2727 | 1589 | 2974 | 1489 | 3667 | 1459 | 3182 | 914 |
| *Notes:* Table reports parameter estimates and standard errors (in parentheses) from binned regressions on WHO5 index on log(local infection rate) by survey round, gender and living arrangements fixed effects. All models control for weather as described under ‘Statistical Procedures’. Population weights from Statistics Denmark were applied.  º denotes responses prior to the first lockdown. ^***^ p<0.01, ^**^ p<0.05, ^*^ p<0.1 (two-tailed tests). | | | | | | | | | | | |

**Appendix C**

Replication code.

The do-files are structured around a master.do which calls do-files for each step of the data processing and analyses. For simplicity, lines of asterixis separate do-files.

< do-files begin >

***************************************************************************************

///////////////////////////////////////////////////////////////////////////////

// Title: Stress/Depression Across the COVID-19 Pandemic in Denmark

// Authors: BLINDED FOR REVIEW

// Master: Prepares data and runs all results

// Output placed in /output and in R/graphs folders

////////////////////////////////////////////////////////////////////////////////

** preamble

clear all

version 16.1

set more off

set graphics on

version 16

set scheme plottigblind

** Paths to the different directories

global yyy y:/data/workdata/707676/BLINDED FOR REVIEW/

global dd100 e:/data/rawdata/707676

*global temp y:/data/workdata/707676/temp

global xxx1 Y:/Data/Workdata707676/BLINDED FOR REVIEW

global xxx Y:/Data/Workdata/707676/BLINDED FOR REVIEW

// Data cleaning and preparation

do $xxx/do/01_data_clean_and_prep.do // survey data and data on infections and weather

// Descriptive statistics

do $xxx/do/02_desct_stat01.do

// Analysis

do $xxx/do/03_analysis01.do

do $xxx/do/03_analysis02.do

do $xxx/do/03_analysis03.do

// R&R

do $xxx/do/r&r.do

***************************************************************************************

///////////////////////////////////////////////////////////////////////////////

// Title: Stress/Depression Across the COVID-19 Pandemic in Denmark

// Authors: BLINDED FOR REVIEW

// 01_data_clean_and_prep: this do-file clean raw data and prepare it for the analysis

////////////////////////////////////////////////////////////////////////////////

cd $yyy

/// Defining globals for each survey wave to only use variables of interest

#delimit;

gl vars_0 pnr int_dato round koen aldergrp region opr_type famtype HjembBoern pervgt pervgt_1 pervgt_2 Helbred Fysik F1 F2 F3 F4 F5 C1 C2 C3 C4 C5 Fam Ven Kolleg Nabo Intern;

gl vars_1 pnr int_dato round Helbred Fysik F1 F2 F3 F4 F5 C1 C2 C3 C4 C5 pervgt H* S1* S2* L1_1* L1_2* Fam Ven Kolleg Nabo Intern;

gl vars_2 pnr int_dato round koen aldergrp region opr_type famtype pervgt HjembBoern Helbred Fysik F1 F2 F3 F4 F5 C1 C2 C3 C4 C5 fam Ven Kolleg Nabo Inter;

gl vars_3 pnr int_dato round Helbred Fysik F1 F2 F3 F4 F5 C1 C2 C3 C4 pervgt C5 H* S1* S2* L1_1* L1_2* Fam Ven Kolleg Nabo Intern;

gl vars_4 pnr int_dato round koen aldergrp region opr_type famtype pervgt HjembBoern Helbred Fysik F1 F2 F3 F4 F5 C1 C2 C3 C4 C5 Fam Ven Kolleg Nabo Intern;

gl vars_5 pnr int_dato round Helbred Fysik F1 F2 F3 F4 F5 C1 C2 C3 C4 pervgt C5 H* S1* S2* L1_1* L1_2* fam Ven Kolleg Nabo Intern;

gl vars_6 pnr int_dato round koen aldergrp region opr_type famtype pervgt HjembBoern Helbred Fysik F1 F2 F3 F4 F5 C1 C2 C3 C4 C5 Fam Ven Kolleg Nabo Intern;

gl vars_7 pnr int_dato round Helbred Fysik F1 F2 F3 F4 F5 C1 C2 C3 C4 pervgt C5 H* S1* S2* L1_1* L1_2* fam Ven Kolleg Nabo Intern;

gl vars_8 pnr int_dato round koen aldergrp region opr_type famtype pervgt HjembBoern Helbred Fysik F1 F2 F3 F4 F5 C1 C2 C3 C4 C5 H* S1* S2* L1_1* L1_2* Fam Ven Kolleg Nabo Intern;

gl vars_9 pnr int_dato round Helbred Fysik F1 F2 F3 F4 F5 C1 C2 C3 pervgt C4 C5 H* S1* S2* L1_1* L1_2* fam Ven Kolleg Nabo Inter;

gl consis koen aldergrp region opr_type famtype HjembBoern Helbred Fysik F1 F2 F3 F4 F5 C1 C2 C3 C4 C5 Fam Ven Kolleg Nabo Intern;

#delimit cr

/// Data cleaning

/*Note: This part cleans the data, at each survey wave, deletes very late responses, and generates variables of interest. To make it more effciient and minimize the risk of coding-errors we use a loop */

local latest = 9

foreach round of numlist 0/`latest' {

disp in red `round'

if "`round'" == "0" local data "crosssection_March_2020_svar.dta"

if "`round'" == "1" local data "panel_July_2020_svar"

if "`round'" == "2" local data "crosssection_September_2020_svar.dta"

if "`round'" == "3" local data "panel_November_2020_svar.dta"

if "`round'" == "4" local data "crosssection_March_2021_svar.dta"

if "`round'" == "5" local data "panel_March_2021_svar.dta"

if "`round'" == "6" local data "crosssection_September_2021_svar.dta"

if "`round'" == "7" local data "panel_November_2021_svar.dta"

if "`round'" == "8" local data "crosssection_March_2022_svar.dta"

if "`round'" == "9" local data "panel_March_2022_svar.dta"

disp in red "`data'"

use data/`data', clear

*** Rename variables for consistency

if "`round'" == "4" {

rename Fam fam

drop if int_dato>mdy(4,24,2021)

drop if int_dato>mdy(03,26,2021) & int_dato<mdy(04,05,2021)

drop if int_resultat==2

}

if "`round'" == "5" {

drop if int_dato>mdy(03,26,2021) & int_dato<mdy(04,05,2021)

}

if "`round'" == "6" {

rename Fam fam

}

if "`round'" == "8" {

rename Fam fam

drop if int_dato>mdy(4,24,2022)

drop if int_dato>mdy(03,26,2022) & int_dato<mdy(04,05,2022)

drop if int_resultat==2

}

if "`round'" == "9" {

drop if int_dato>mdy(03,26,2022) & int_dato<mdy(04,05,2022)

}

if "`round'" > "0" {

rename (fam Inter) (Fam Intern)

}

*** Generate survey round variable

gen round = `round'

*** Keeping relevant variables

keep ${vars_`round'}

*** Checking code consistency

*fre $consis

*** Check reliability using Chronbach's alpha

alpha F1 F2 F3 F4 F5

disp in red "WHO-5 - Chronbach's alpha = `r(alpha)'"

** Generating/re-coding variables

*** WHO5

gen WHO5=(F1+F2+F3+F4+F5)*4

**** WHO5 <= 50

gen WHO5_50 = WHO5 <= 50 /*Increased risk of depression*/

label var WHO5_50 "Increased risk of depression"

** Saving dataset

if "`round'" == "0" {

/* NOTE: This dataset contains respondants' socioeconomics characteristics during survey's first round. Therefore, this dataset should be merged with subsequent rounds for robustness check

*/

preserve

keep pnr koen aldergrp region opr_type famtype HjembBoern

tempfile socio_march

save `socio_march', replace

count

restore

}

if "`round'" == "0" local date "March_20"

if "`round'" == "1" local date "July_20"

if "`round'" == "2" local date "Sept_20"

if "`round'" == "3" local date "Nov_20"

if "`round'" == "4" local date "March_21_c"

if "`round'" == "5" local date "March_21_p"

if "`round'" == "6" local date "Sept_21"

if "`round'" == "7" local date "Nov_21"

if "`round'" == "8" local date "March_22_c"

if "`round'" == "9" local date "March_22_p"

tempfile `date'

save "`date'", replace

}

use March_20, clear

foreach data in July_20 Sept_20 Nov_20 March_21_c March_21_p Sept_21 Nov_21 ///

March_22_c March_22_p {

append using `data'

** Balanced panel

tempvar g

egen `g' = rowmiss(pnr WHO5)

tempvar h

bys pnr: egen `h'=max(`g')

drop if `g'>0

}

** Re-code survey round

/// Note: This is following the Survey-panel structure

gen round_p = .

replace round_p = 1 if round == 0

replace round_p = 2 if round == 1

replace round_p = 3 if round == 3

replace round_p = 4 if round == 5

replace round_p = 5 if round == 7

replace round_p = 6 if round == 9

label define round_p 1 "March-20" 2 "July-20" 3 "Nov-20" 4 "March-21" ///

5 "Nov-21" 6 "March-22"

label values round_p round_p

label var round_p "Rounds Panel"

** Gender

tab koen, gen(gen_)

rename gen_1 Male

rename gen_2 Female

** Regions

tab region, gen(junk)

rename junk1 NorthernJutland

rename junk2 CentralJutland

rename junk3 SouthernJutland

rename junk4 Capitol

rename junk5 Zealand

** Children/no children at home

gen Children=HjembBoern==1

gen NoChildren=HjembBoern==2

** Matching municipality where the person lived at the moment of each survey (from population register)

foreach y in 2019 2020 {

preserve

use pnr familie_id kom bop_vfra reg foed_dag using $dd100/bef12_`y'.dta, clear

rename (bop_vfra kom reg) (bop_vfra`y' kom`y' reg`y')

recast long bop_vfra`y'

tempfile bef`y'

save `bef`y'', replace

restore

}

** Merging with population registers

destring pnr, replace

merge m:1 pnr using `bef2019', keep(3) nogen

merge m:1 pnr using `bef2020', keep(3) nogen

drop region

foreach v in kom reg {

if "`v'" == "kom" local var "kom"

if "`v'" == "reg" local var "region"

tempvar help

gen `help' = 0

replace `help' = 1 if `v'2019!=`v'2020

gen `var' = `v'2019 if bop_vfra2019==bop_vfra2020

replace `var' = `v'2019 if int_dato<=bop_vfra2020

replace `var' = `v'2020 if int_dato>bop_vfra2020

drop `v'2019 `v'2020

}

destring region, replace

** Keeping and ordering variables

keep pnr int_dato round round_p koen aldergrp foed_dag region ///

opr_type famtype HjembBoern ///

pervgt pervgt_1 pervgt_2 Helbred Fysik F1 F2 F3 F4 F5 ///

C1 C2 C3 C4 C5 Fam Ven Kolleg Nabo Intern WHO5 WHO5_50 ///

Male Female Children kom NorthernJutland CentralJutland ///

SouthernJutland Capitol Zealand

order pnr int_dato round round_p koen alder aldergrp foed_dag region ///

kom opr_type famtype HjembBoern ///

pervgt pervgt_1 pervgt_2 Helbred Fysik F1 F2 F3 F4 F5 ///

C1 C2 C3 C4 C5 Fam Ven Kolleg Nabo Intern WHO5 WHO5_50 ///

Male Female Children ///

NorthernJutland CentralJutland SouthernJutland Capitol Zealand

/// This part applies to the panel NOT TO CROSS-SECTION

local num = 5 /*Change this local to the latest survey round (PANEL) */

tempvar junk

tab round_p, gen(`junk'_)

*** Sort observations

foreach r of numlist 1/`num' {

bys pnr: egen in_round`r' = max(`junk'_`r' == 1)

}

*** Select one obs per "pnr"

egen flag = tag(pnr)

*** Dropping irrelevant variables

drop __00000*

**** labeling region

label values region .

label define region 81 "Northern Jutland" 82 "Central Jutland" ///

83 "Southern Jutland" 84 "Capitol" 85 "Zealand"

label values region region

**** labeling age group variable

label values aldergrp .

label define aldergrp 1 "18-29" 2 "30-39" 3 "40-49" 4 "50-59" 5 "60-69" 6 "70-79"

label values aldergrp aldergrp

**** Labeling rounds

/*Note: Variable round includes cross-sectional data and panel data */

rename round help

gen round = .

local junk = 0

foreach num of numlist 1/10 {

replace round = `num' if help == `junk'

local junk = `junk' + 1

}

label define round 1 "March_20" 2 "July_20" 3 "Sept_20" 4 "Nov_20" 5 "March_21_c" ///

6 "March_21_p" 7 "Sept_21" 8 "Nov_21" 9 "March_22_c" 10 "March_22_p"

label values round round

drop help

****** Defining pre-lockdown (first lockdown)

rename round wave

gen round = .

replace round = 0 if int_dato<=mdy(03,11,2020)

replace round = 1 if int_dato>mdy(03,11,2020) & wave==1

foreach r of numlist 2/10 {

replace round = `r' if wave == `r'

}

//// Including weather data from DMI

set obs `=_N+1'

replace int_dato = mdy(03,01,2020) if int_dato==.

set obs `=_N+1'

replace int_dato = mdy(07,01,2020) if int_dato==.

set obs `=_N+1'

replace int_dato = mdy(08,31,2020) if int_dato==.

set obs `=_N+1'

replace int_dato = mdy(11,03,2020) if int_dato==.

set obs `=_N+1'

replace int_dato = mdy(03,02,2021) if int_dato==.

set obs `=_N+1'

replace int_dato = mdy(04,04,2021) if int_dato==.

set obs `=_N+1'

replace int_dato = mdy(09,13,2021) if int_dato==.

set obs `=_N+1'

replace int_dato = mdy(11,01,2021) if int_dato==.

set obs `=_N+1'

replace int_dato = mdy(12,04,2021) if int_dato==.

set obs `=_N+1'

replace int_dato = mdy(12,24,2021) if int_dato==.

set obs `=_N+1'

replace int_dato = mdy(02,28,2022) if int_dato==.

set obs `=_N+1'

replace int_dato = mdy(04,04,2022) if int_dato==.

gen avg_temp = .

{

replace avg_temp = 4.9 if int_dato == mdy(03,01,2020)

replace avg_temp = 4.3 if int_dato == mdy(03,02,2020)

replace avg_temp = 4.0 if int_dato == mdy(03,03,2020)

replace avg_temp = 3.5 if int_dato == mdy(03,04,2020)

replace avg_temp = 2.9 if int_dato == mdy(03,05,2020)

replace avg_temp = 3.0 if int_dato == mdy(03,06,2020)

replace avg_temp = 3.0 if int_dato == mdy(03,07,2020)

replace avg_temp = 6.4 if int_dato == mdy(03,08,2020)

replace avg_temp = 6.4 if int_dato == mdy(03,09,2020)

replace avg_temp = 5.9 if int_dato == mdy(03,10,2020)

replace avg_temp = 6.8 if int_dato == mdy(03,11,2020)

replace avg_temp = 5.6 if int_dato == mdy(03,12,2020)

replace avg_temp = 3.9 if int_dato == mdy(03,13,2020)

replace avg_temp = 2 if int_dato == mdy(03,14,2020)

replace avg_temp = 6.4 if int_dato == mdy(03,15,2020)

replace avg_temp = 7.0 if int_dato == mdy(03,16,2020)

replace avg_temp = 6.7 if int_dato == mdy(03,17,2020)

replace avg_temp = 7.3 if int_dato == mdy(03,18,2020)

replace avg_temp = 5.7 if int_dato == mdy(03,19,2020)

replace avg_temp = 4.3 if int_dato == mdy(03,20,2020)

replace avg_temp = 2.5 if int_dato == mdy(03,21,2020)

replace avg_temp = 2.0 if int_dato == mdy(03,22,2020)

replace avg_temp = 2.3 if int_dato == mdy(03,23,2020)

replace avg_temp = 3.7 if int_dato == mdy(03,24,2020)

replace avg_temp = 4.6 if int_dato == mdy(03,25,2020)

replace avg_temp = 4.1 if int_dato == mdy(03,26,2020)

replace avg_temp = 4.4 if int_dato == mdy(03,27,2020)

replace avg_temp = 4.9 if int_dato == mdy(03,28,2020)

replace avg_temp = 2.4 if int_dato == mdy(03,29,2020)

replace avg_temp = 3.7 if int_dato == mdy(03,30,2020)

replace avg_temp = 2.7 if int_dato == mdy(03,31,2020)

replace avg_temp = 5.9 if int_dato == mdy(04,01,2020)

replace avg_temp = 5.9 if int_dato == mdy(04,02,2020)

replace avg_temp = 4.3 if int_dato == mdy(04,03,2020)

replace avg_temp = 4.9 if int_dato == mdy(04,04,2020)

replace avg_temp = 8.2 if int_dato == mdy(04,05,2020)

replace avg_temp = 10.4 if int_dato == mdy(04,06,2020)

replace avg_temp = 9.7 if int_dato == mdy(04,07,2020)

replace avg_temp = 9.2 if int_dato == mdy(04,08,2020)

replace avg_temp = 5.4 if int_dato == mdy(04,11,2020)

replace avg_temp = 9.7 if int_dato == mdy(04,13,2020)

replace avg_temp = 15.5 if int_dato == mdy(07,01,2020)

replace avg_temp = 14.9 if int_dato == mdy(07,02,2020)

replace avg_temp = 13.2 if int_dato == mdy(07,03,2020)

replace avg_temp = 11.7 if int_dato == mdy(07,04,2020)

replace avg_temp = 15.9 if int_dato == mdy(07,05,2020)

replace avg_temp = 13.3 if int_dato == mdy(07,06,2020)

replace avg_temp = 12.5 if int_dato == mdy(07,07,2020)

replace avg_temp = 12.4 if int_dato == mdy(07,08,2020)

replace avg_temp = 12.7 if int_dato == mdy(07,09,2020)

replace avg_temp = 11.9 if int_dato == mdy(07,10,2020)

replace avg_temp = 13.3 if int_dato == mdy(07,11,2020)

replace avg_temp = 13.5 if int_dato == mdy(07,12,2020)

replace avg_temp = 14.4 if int_dato == mdy(07,13,2020)

replace avg_temp = 14.7 if int_dato == mdy(07,14,2020)

replace avg_temp = 14.4 if int_dato == mdy(07,15,2020)

replace avg_temp = 14.9 if int_dato == mdy(07,16,2020)

replace avg_temp = 16.0 if int_dato == mdy(07,17,2020)

replace avg_temp = 17.9 if int_dato == mdy(07,18,2020)

replace avg_temp = 18.3 if int_dato == mdy(07,19,2020)

replace avg_temp = 15.1 if int_dato == mdy(07,20,2020)

replace avg_temp = 14.2 if int_dato == mdy(07,21,2020)

replace avg_temp = 13.9 if int_dato == mdy(07,22,2020)

replace avg_temp = 13.8 if int_dato == mdy(07,23,2020)

replace avg_temp = 15.6 if int_dato == mdy(07,24,2020)

replace avg_temp = 15.7 if int_dato == mdy(07,25,2020)

replace avg_temp = 16.9 if int_dato == mdy(07,26,2020)

replace avg_temp = 16.4 if int_dato == mdy(07,27,2020)

replace avg_temp = 16.3 if int_dato == mdy(07,28,2020)

replace avg_temp = 13.9 if int_dato == mdy(07,29,2020)

replace avg_temp = 14.3 if int_dato == mdy(07,30,2020)

replace avg_temp = 16.1 if int_dato == mdy(07,31,2020)

replace avg_temp = 18.1 if int_dato == mdy(08,01,2020)

replace avg_temp = 14.3 if int_dato == mdy(08,31,2020)

replace avg_temp = 13.6 if int_dato == mdy(09,01,2020)

replace avg_temp = 14.8 if int_dato == mdy(09,02,2020)

replace avg_temp = 14.0 if int_dato == mdy(09,03,2020)

replace avg_temp = 15.9 if int_dato == mdy(09,04,2020)

replace avg_temp = 13.7 if int_dato == mdy(09,05,2020)

replace avg_temp = 12.6 if int_dato == mdy(09,06,2020)

replace avg_temp = 13.3 if int_dato == mdy(09,07,2020)

replace avg_temp = 16.0 if int_dato == mdy(09,08,2020)

replace avg_temp = 15.7 if int_dato == mdy(09,09,2020)

replace avg_temp = 12.9 if int_dato == mdy(09,10,2020)

replace avg_temp = 13.1 if int_dato == mdy(09,11,2020)

replace avg_temp = 14.3 if int_dato == mdy(09,12,2020)

replace avg_temp = 15.1 if int_dato == mdy(09,13,2020)

replace avg_temp = 17.5 if int_dato == mdy(09,14,2020)

replace avg_temp = 16.8 if int_dato == mdy(09,15,2020)

replace avg_temp = 14.3 if int_dato == mdy(09,16,2020)

replace avg_temp = 11.0 if int_dato == mdy(09,17,2020)

replace avg_temp = 10.6 if int_dato == mdy(09,18,2020)

replace avg_temp = 12.1 if int_dato == mdy(09,19,2020)

replace avg_temp = 12.1 if int_dato == mdy(09,20,2020)

replace avg_temp = 12.6 if int_dato == mdy(09,21,2020)

replace avg_temp = 13.1 if int_dato == mdy(09,22,2020)

replace avg_temp = 14.8 if int_dato == mdy(09,23,2020)

replace avg_temp = 14.9 if int_dato == mdy(09,24,2020)

replace avg_temp = 12.5 if int_dato == mdy(09,25,2020)

replace avg_temp = 12.7 if int_dato == mdy(09,26,2020)

replace avg_temp = 16.1 if int_dato == mdy(09,27,2020)

replace avg_temp = 14.4 if int_dato == mdy(09,28,2020)

replace avg_temp = 13.4 if int_dato == mdy(09,29,2020)

replace avg_temp = 13.9 if int_dato == mdy(09,30,2020)

replace avg_temp = 9.6 if int_dato == mdy(11,03,2020)

replace avg_temp = 8.5 if int_dato == mdy(11,04,2020)

replace avg_temp = 10.1 if int_dato == mdy(11,05,2020)

replace avg_temp = 11.1 if int_dato == mdy(11,06,2020)

replace avg_temp = 8.7 if int_dato == mdy(11,07,2020)

replace avg_temp = 6.5 if int_dato == mdy(11,08,2020)

replace avg_temp = 7.5 if int_dato == mdy(11,09,2020)

replace avg_temp = 7.2 if int_dato == mdy(11,10,2020)

replace avg_temp = 5.8 if int_dato == mdy(11,11,2020)

replace avg_temp = 7.4 if int_dato == mdy(11,12,2020)

replace avg_temp = 8.1 if int_dato == mdy(11,13,2020)

replace avg_temp = 8.3 if int_dato == mdy(11,14,2020)

replace avg_temp = 11.3 if int_dato == mdy(11,15,2020)

replace avg_temp = 9.6 if int_dato == mdy(11,16,2020)

replace avg_temp = 10.4 if int_dato == mdy(11,17,2020)

replace avg_temp = 11.2 if int_dato == mdy(11,18,2020)

replace avg_temp = 7.0 if int_dato == mdy(11,19,2020)

replace avg_temp = 4.1 if int_dato == mdy(11,20,2020)

replace avg_temp = 7.8 if int_dato == mdy(11,21,2020)

replace avg_temp = 8.0 if int_dato == mdy(11,22,2020)

replace avg_temp = 7.2 if int_dato == mdy(11,23,2020)

replace avg_temp = 8.7 if int_dato == mdy(11,24,2020)

replace avg_temp = 7.0 if int_dato == mdy(11,25,2020)

replace avg_temp = 7.0 if int_dato == mdy(11,26,2020)

replace avg_temp = 2.9 if int_dato == mdy(11,27,2020)

replace avg_temp = 1.0 if int_dato == mdy(11,28,2020)

replace avg_temp = 1.1 if int_dato == mdy(11,29,2020)

replace avg_temp = 1.7 if int_dato == mdy(11,30,2020)

replace avg_temp = 2.0 if int_dato == mdy(03,02,2021)

replace avg_temp = 1.1 if int_dato == mdy(03,03,2021)

replace avg_temp = 1.7 if int_dato == mdy(03,04,2021)

replace avg_temp = 0.3 if int_dato == mdy(03,05,2021)

replace avg_temp = 2.7 if int_dato == mdy(03,06,2021)

replace avg_temp = 2.7 if int_dato == mdy(03,07,2021)

replace avg_temp = -0.4 if int_dato == mdy(03,08,2021)

replace avg_temp = 0.0 if int_dato == mdy(03,09,2021)

replace avg_temp = 0.9 if int_dato == mdy(03,10,2021)

replace avg_temp = 4.6 if int_dato == mdy(03,11,2021)

replace avg_temp = 4.6 if int_dato == mdy(03,12,2021)

replace avg_temp = 3.2 if int_dato == mdy(03,13,2021)

replace avg_temp = 4.1 if int_dato == mdy(03,14,2021)

replace avg_temp = 4.0 if int_dato == mdy(03,15,2021)

replace avg_temp = 2.9 if int_dato == mdy(03,16,2021)

replace avg_temp = 2.6 if int_dato == mdy(03,17,2021)

replace avg_temp = 2.1 if int_dato == mdy(03,18,2021)

replace avg_temp = 0.8 if int_dato == mdy(03,19,2021)

replace avg_temp = 3.1 if int_dato == mdy(03,20,2021)

replace avg_temp = 5.0 if int_dato == mdy(03,21,2021)

replace avg_temp = 4.2 if int_dato == mdy(03,22,2021)

replace avg_temp = 5.3 if int_dato == mdy(03,23,2021)

replace avg_temp = 6.7 if int_dato == mdy(03,24,2021)

replace avg_temp = 6.1 if int_dato == mdy(03,25,2021)

replace avg_temp = 7.5 if int_dato == mdy(03,26,2021)

replace avg_temp = 5.0 if int_dato == mdy(04,04,2021)

replace avg_temp = 2.8 if int_dato == mdy(04,05,2021)

replace avg_temp = 3.2 if int_dato == mdy(04,06,2021)

replace avg_temp = 3.8 if int_dato == mdy(04,07,2021)

replace avg_temp = 3.8 if int_dato == mdy(04,08,2021)

replace avg_temp = 5.3 if int_dato == mdy(04,09,2021)

replace avg_temp = 3.6 if int_dato == mdy(04,10,2021)

replace avg_temp = 2.3 if int_dato == mdy(04,11,2021)

replace avg_temp = 3.6 if int_dato == mdy(04,12,2021)

replace avg_temp = 4.3 if int_dato == mdy(04,13,2021)

replace avg_temp = 4.1 if int_dato == mdy(04,14,2021)

replace avg_temp = 4.8 if int_dato == mdy(04,15,2021)

replace avg_temp = 5.9 if int_dato == mdy(04,16,2021)

replace avg_temp = 7.9 if int_dato == mdy(04,17,2021)

replace avg_temp = 9.4 if int_dato == mdy(04,18,2021)

replace avg_temp = 10.1 if int_dato == mdy(04,19,2021)

replace avg_temp = 9.6 if int_dato == mdy(04,20,2021)

replace avg_temp = 7.0 if int_dato == mdy(04,21,2021)

replace avg_temp = 6.7 if int_dato == mdy(04,22,2021)

replace avg_temp = 6.5 if int_dato == mdy(04,23,2021)

replace avg_temp = 5.5 if int_dato == mdy(04,24,2021)

replace avg_temp = 15.1 if int_dato == mdy(09,13,2021)

replace avg_temp = 15.0 if int_dato == mdy(09,14,2021)

replace avg_temp = 14.6 if int_dato == mdy(09,15,2021)

replace avg_temp = 15.1 if int_dato == mdy(09,16,2021)

replace avg_temp = 12.6 if int_dato == mdy(09,17,2021)

replace avg_temp = 13.5 if int_dato == mdy(09,18,2021)

replace avg_temp = 11.7 if int_dato == mdy(09,19,2021)

replace avg_temp = 11.0 if int_dato == mdy(09,20,2021)

replace avg_temp = 11.9 if int_dato == mdy(09,21,2021)

replace avg_temp = 14.3 if int_dato == mdy(09,22,2021)

replace avg_temp = 14.0 if int_dato == mdy(09,23,2021)

replace avg_temp = 13.7 if int_dato == mdy(09,24,2021)

replace avg_temp = 14.3 if int_dato == mdy(09,25,2021)

replace avg_temp = 13.7 if int_dato == mdy(09,26,2021)

replace avg_temp = 15.7 if int_dato == mdy(09,27,2021)

replace avg_temp = 13.6 if int_dato == mdy(09,28,2021)

replace avg_temp = 12.3 if int_dato == mdy(09,29,2021)

replace avg_temp = 11.4 if int_dato == mdy(09,30,2021)

replace avg_temp = 12.6 if int_dato == mdy(10,01,2021)

replace avg_temp = 12.9 if int_dato == mdy(10,02,2021)

replace avg_temp = 14.6 if int_dato == mdy(10,03,2021)

replace avg_temp = 12.6 if int_dato == mdy(10,04,2021)

replace avg_temp = 11.7 if int_dato == mdy(10,05,2021)

replace avg_temp = 11.9 if int_dato == mdy(10,06,2021)

replace avg_temp = 10.7 if int_dato == mdy(10,07,2021)

replace avg_temp = 10.5 if int_dato == mdy(10,08,2021)

replace avg_temp = 10.3 if int_dato == mdy(10,09,2021)

replace avg_temp = 9.2 if int_dato == mdy(10,10,2021)

replace avg_temp = 10.2 if int_dato == mdy(10,11,2021)

replace avg_temp = 7.9 if int_dato == mdy(10,12,2021)

replace avg_temp = 7.6 if int_dato == mdy(10,13,2021)

replace avg_temp = 11.4 if int_dato == mdy(10,14,2021)

replace avg_temp = 10.6 if int_dato == mdy(10,15,2021)

replace avg_temp = 8.9 if int_dato == mdy(10,16,2021)

replace avg_temp = 8.7 if int_dato == mdy(10,17,2021)

replace avg_temp = 9.5 if int_dato == mdy(10,18,2021)

replace avg_temp = 11.8 if int_dato == mdy(10,19,2021)

replace avg_temp = 13.8 if int_dato == mdy(10,20,2021)

replace avg_temp = 9.2 if int_dato == mdy(10,21,2021)

replace avg_temp = 7.5 if int_dato == mdy(10,22,2021)

replace avg_temp = 8.1 if int_dato == mdy(10,23,2021)

replace avg_temp = 8.9 if int_dato == mdy(10,24,2021)

replace avg_temp = 8.4 if int_dato == mdy(10,25,2021)

replace avg_temp = 10.2 if int_dato == mdy(10,26,2021)

replace avg_temp = 13.0 if int_dato == mdy(10,27,2021)

replace avg_temp = 12.4 if int_dato == mdy(10,28,2021)

replace avg_temp = 10.5 if int_dato == mdy(10,29,2021)

replace avg_temp = 10.8 if int_dato == mdy(10,30,2021)

replace avg_temp = 10.8 if int_dato == mdy(10,31,2021)

replace avg_temp = 10.8 if int_dato == mdy(11,01,2021)

replace avg_temp = 8.0 if int_dato == mdy(11,02,2021)

replace avg_temp = 5.5 if int_dato == mdy(11,03,2021)

replace avg_temp = 5.7 if int_dato == mdy(11,04,2021)

replace avg_temp = 8.1 if int_dato == mdy(11,05,2021)

replace avg_temp = 10.0 if int_dato == mdy(11,06,2021)

replace avg_temp = 8.5 if int_dato == mdy(11,07,2021)

replace avg_temp = 6.7 if int_dato == mdy(11,08,2021)

replace avg_temp = 9.1 if int_dato == mdy(11,09,2021)

replace avg_temp = 10.2 if int_dato == mdy(11,10,2021)

replace avg_temp = 8.7 if int_dato == mdy(11,11,2021)

replace avg_temp = 7.6 if int_dato == mdy(11,12,2021)

replace avg_temp = 7.4 if int_dato == mdy(11,13,2021)

replace avg_temp = 7.7 if int_dato == mdy(11,14,2021)

replace avg_temp = 6.9 if int_dato == mdy(11,15,2021)

replace avg_temp = 5.3 if int_dato == mdy(11,16,2021)

replace avg_temp = 6.0 if int_dato == mdy(11,17,2021)

replace avg_temp = 9.4 if int_dato == mdy(11,18,2021)

replace avg_temp = 11.3 if int_dato == mdy(11,19,2021)

replace avg_temp = 10.0 if int_dato == mdy(11,20,2021)

replace avg_temp = 5.4 if int_dato == mdy(11,21,2021)

replace avg_temp = 3.7 if int_dato == mdy(11,22,2021)

replace avg_temp = 7.1 if int_dato == mdy(11,23,2021)

replace avg_temp = 7.4 if int_dato == mdy(11,24,2021)

replace avg_temp = 5.8 if int_dato == mdy(11,25,2021)

replace avg_temp = 3.1 if int_dato == mdy(11,26,2021)

replace avg_temp = 2.9 if int_dato == mdy(11,27,2021)

replace avg_temp = 2.0 if int_dato == mdy(11,28,2021)

replace avg_temp = 0.5 if int_dato == mdy(11,29,2021)

replace avg_temp = 2.1 if int_dato == mdy(11,30,2021)

replace avg_temp = 1.9 if int_dato == mdy(12,01,2021)

replace avg_temp = -0.1 if int_dato == mdy(12,02,2021)

replace avg_temp = 1.5 if int_dato == mdy(12,03,2021)

replace avg_temp = 7.6 if int_dato == mdy(12,04,2021)

replace avg_temp = 1.1 if int_dato == mdy(12,05,2021)

replace avg_temp = -0.1 if int_dato == mdy(12,06,2021)

replace avg_temp = 1.2 if int_dato == mdy(12,07,2021)

replace avg_temp = 1.5 if int_dato == mdy(12,08,2021)

replace avg_temp = 1.1 if int_dato == mdy(12,09,2021)

replace avg_temp = 1.4 if int_dato == mdy(12,10,2021)

replace avg_temp = 0.8 if int_dato == mdy(12,11,2021)

replace avg_temp = 3.3 if int_dato == mdy(12,12,2021)

replace avg_temp = 6.8 if int_dato == mdy(12,13,2021)

replace avg_temp = 5.7 if int_dato == mdy(12,14,2021)

replace avg_temp = 8.0 if int_dato == mdy(12,15,2021)

replace avg_temp = 6.5 if int_dato == mdy(12,16,2021)

replace avg_temp = 5.9 if int_dato == mdy(12,17,2021)

replace avg_temp = 6.6 if int_dato == mdy(12,18,2021)

replace avg_temp = 5.2 if int_dato == mdy(12,19,2021)

replace avg_temp = 0.8 if int_dato == mdy(12,20,2021)

replace avg_temp = -0.5 if int_dato == mdy(12,21,2021)

replace avg_temp = 1.6 if int_dato == mdy(12,22,2021)

replace avg_temp = -0.1 if int_dato == mdy(12,23,2021)

replace avg_temp = -3.0 if int_dato == mdy(12,24,2021)

replace avg_temp = -4-4 if int_dato == mdy(12,25,2021)

replace avg_temp = -4.6 if int_dato == mdy(12,26,2021)

replace avg_temp = -2.3 if int_dato == mdy(12,27,2021)

replace avg_temp = 0.4 if int_dato == mdy(12,28,2021)

replace avg_temp = 1.2 if int_dato == mdy(12,29,2021)

replace avg_temp = 6.3 if int_dato == mdy(12,30,2021)

}

replace avg_temp = 2.9 if int_dato == mdy(02,28,2022)

replace avg_temp = 2.2 if int_dato == mdy(03,01,2022)

replace avg_temp = 1.6 if int_dato == mdy(03,02,2022)

replace avg_temp = 1.4 if int_dato == mdy(03,03,2022)

replace avg_temp = 0.9 if int_dato == mdy(03,04,2022)

replace avg_temp = 0.3 if int_dato == mdy(03,05,2022)

replace avg_temp = 1.4 if int_dato == mdy(03,06,2022)

replace avg_temp = 1.5 if int_dato == mdy(03,07,2022)

replace avg_temp = 2.1 if int_dato == mdy(03,08,2022)

replace avg_temp = 1.4 if int_dato == mdy(03,09,2022)

replace avg_temp = 3.3 if int_dato == mdy(03,10,2022)

replace avg_temp = 3.3 if int_dato == mdy(03,11,2022)

replace avg_temp = 4.0 if int_dato == mdy(03,12,2022)

replace avg_temp = 4.6 if int_dato == mdy(03,13,2022)

replace avg_temp = 5.1 if int_dato == mdy(03,14,2022)

replace avg_temp = 4.4 if int_dato == mdy(03,15,2022)

replace avg_temp = 2.8 if int_dato == mdy(03,16,2022)

replace avg_temp = 4.5 if int_dato == mdy(03,17,2022)

replace avg_temp = 5.4 if int_dato == mdy(03,18,2022)

replace avg_temp = 4.2 if int_dato == mdy(03,19,2022)

replace avg_temp = 4.1 if int_dato == mdy(03,20,2022)

replace avg_temp = 5.5 if int_dato == mdy(03,21,2022)

replace avg_temp = 5.4 if int_dato == mdy(03,22,2022)

replace avg_temp = 5.2 if int_dato == mdy(03,23,2022)

replace avg_temp = 6.3 if int_dato == mdy(03,24,2022)

replace avg_temp = 8.0 if int_dato == mdy(03,25,2022)

replace avg_temp = 8.1 if int_dato == mdy(03,26,2022)

replace avg_temp = 5.0 if int_dato == mdy(04,04,2022)

replace avg_temp = 3.7 if int_dato == mdy(04,05,2022)

replace avg_temp = 4.6 if int_dato == mdy(04,06,2022)

replace avg_temp = 5.6 if int_dato == mdy(04,07,2022)

replace avg_temp = 3.6 if int_dato == mdy(04,08,2022)

replace avg_temp = 4.5 if int_dato == mdy(04,09,2022)

replace avg_temp = 4.5 if int_dato == mdy(04,10,2022)

replace avg_temp = 4.6 if int_dato == mdy(04,11,2022)

replace avg_temp = 5.0 if int_dato == mdy(04,12,2022)

replace avg_temp = 9.3 if int_dato == mdy(04,13,2022)

replace avg_temp = 8.1 if int_dato == mdy(04,14,2022)

replace avg_temp = 6.3 if int_dato == mdy(04,15,2022)

replace avg_temp = 6.3 if int_dato == mdy(04,16,2022)

replace avg_temp = 7.2 if int_dato == mdy(04,17,2022)

replace avg_temp = 8.1 if int_dato == mdy(04,18,2022)

/// Average Sunligth

gen avg_sun = .

{

replace avg_sun = 2.4 if int_dato == mdy(03,01,2020)

replace avg_sun = 4.7 if int_dato == mdy(03,02,2020)

replace avg_sun = 1.2 if int_dato == mdy(03,03,2020)

replace avg_sun = 4.7 if int_dato == mdy(03,04,2020)

replace avg_sun = 3.9 if int_dato == mdy(03,05,2020)

replace avg_sun = 1.1 if int_dato == mdy(03,06,2020)

replace avg_sun = 4.9 if int_dato == mdy(03,07,2020)

replace avg_sun = 0.4 if int_dato == mdy(03,08,2020)

replace avg_sun = 1.7 if int_dato == mdy(03,09,2020)

replace avg_sun = 0.0 if int_dato == mdy(03,10,2020)

replace avg_sun = 7.5 if int_dato == mdy(03,11,2020)

replace avg_sun = 3.5 if int_dato == mdy(03,12,2020)

replace avg_sun = 3.7 if int_dato == mdy(03,13,2020)

replace avg_sun = 10.0 if int_dato == mdy(03,14,2020)

replace avg_sun = 0.3 if int_dato == mdy(03,15,2020)

replace avg_sun = 3.1 if int_dato == mdy(03,16,2020)

replace avg_sun = 1.3 if int_dato == mdy(03,17,2020)

replace avg_sun = 0.5 if int_dato == mdy(03,18,2020)

replace avg_sun = 7.4 if int_dato == mdy(03,19,2020)

replace avg_sun = 8.7 if int_dato == mdy(03,20,2020)

replace avg_sun = 10.5 if int_dato == mdy(03,21,2020)

replace avg_sun = 8.3 if int_dato == mdy(03,22,2020)

replace avg_sun = 11.1 if int_dato == mdy(03,23,2020)

replace avg_sun = 10.0 if int_dato == mdy(03,24,2020)

replace avg_sun = 11.1 if int_dato == mdy(03,25,2020)

replace avg_sun = 11.3 if int_dato == mdy(03,26,2020)

replace avg_sun = 11.5 if int_dato == mdy(03,27,2020)

replace avg_sun = 8.8 if int_dato == mdy(03,28,2020)

replace avg_sun = 11.2 if int_dato == mdy(03,29,2020)

replace avg_sun = 9.6 if int_dato == mdy(03,30,2020)

replace avg_sun = 7.5 if int_dato == mdy(03,31,2020)

replace avg_sun = 2.0 if int_dato == mdy(04,01,2020)

replace avg_sun = 2.1 if int_dato == mdy(04,02,2020)

replace avg_sun = 8.4 if int_dato == mdy(04,03,2020)

replace avg_sun = 5.6 if int_dato == mdy(04,04,2020)

replace avg_sun = 11.7 if int_dato == mdy(04,05,2020)

replace avg_sun = 12.4 if int_dato == mdy(04,06,2020)

replace avg_sun = 12.2 if int_dato == mdy(04,07,2020)

replace avg_sun = 10.0 if int_dato == mdy(04,08,2020)

replace avg_sun = 2.6 if int_dato == mdy(04,11,2020)

replace avg_sun = 10.1 if int_dato == mdy(04,13,2020)

replace avg_sun = 4.2 if int_dato == mdy(07,01,2020)

replace avg_sun = 9.1 if int_dato == mdy(07,02,2020)

replace avg_sun = 2.7 if int_dato == mdy(07,03,2020)

replace avg_sun = 0.1 if int_dato == mdy(07,04,2020)

replace avg_sun = 3.2 if int_dato == mdy(07,05,2020)

replace avg_sun = 7.1 if int_dato == mdy(07,06,2020)

replace avg_sun = 5.6 if int_dato == mdy(07,07,2020)

replace avg_sun = 7.7 if int_dato == mdy(07,08,2020)

replace avg_sun = 9.4 if int_dato == mdy(07,09,2020)

replace avg_sun = 5.2 if int_dato == mdy(07,10,2020)

replace avg_sun = 9.9 if int_dato == mdy(07,11,2020)

replace avg_sun = 4.6 if int_dato == mdy(07,12,2020)

replace avg_sun = 6.9 if int_dato == mdy(07,13,2020)

replace avg_sun = 2.1 if int_dato == mdy(07,14,2020)

replace avg_sun = 4.1 if int_dato == mdy(07,15,2020)

replace avg_sun = 8.2 if int_dato == mdy(07,16,2020)

replace avg_sun = 9.8 if int_dato == mdy(07,17,2020)

replace avg_sun = 6.0 if int_dato == mdy(07,18,2020)

replace avg_sun = 2.6 if int_dato == mdy(07,19,2020)

replace avg_sun = 8.3 if int_dato == mdy(07,20,2020)

replace avg_sun = 10.1 if int_dato == mdy(07,21,2020)

replace avg_sun = 9.8 if int_dato == mdy(07,22,2020)

replace avg_sun = 4.5 if int_dato == mdy(07,23,2020)

replace avg_sun = 4.5 if int_dato == mdy(07,24,2020)

replace avg_sun = 5.1 if int_dato == mdy(07,25,2020)

replace avg_sun = 3.2 if int_dato == mdy(07,26,2020)

replace avg_sun = 4.6 if int_dato == mdy(07,27,2020)

replace avg_sun = 4.9 if int_dato == mdy(07,28,2020)

replace avg_sun = 2.3 if int_dato == mdy(07,29,2020)

replace avg_sun = 4.4 if int_dato == mdy(07,30,2020)

replace avg_sun = 11.0 if int_dato == mdy(07,31,2020)

replace avg_sun = 10.0 if int_dato == mdy(08,01,2020)

replace avg_sun = 10.2 if int_dato == mdy(08,31,2020)

replace avg_sun = 11.1 if int_dato == mdy(09,01,2020)

replace avg_sun = 9.3 if int_dato == mdy(09,02,2020)

replace avg_sun = 4.0 if int_dato == mdy(09,03,2020)

replace avg_sun = 4.5 if int_dato == mdy(09,04,2020)

replace avg_sun = 5.7 if int_dato == mdy(09,05,2020)

replace avg_sun = 6.9 if int_dato == mdy(09,06,2020)

replace avg_sun = 6.9 if int_dato == mdy(09,07,2020)

replace avg_sun = 5.0 if int_dato == mdy(09,08,2020)

replace avg_sun = 2.5 if int_dato == mdy(09,09,2020)

replace avg_sun = 8.2 if int_dato == mdy(09,10,2020)

replace avg_sun = 2.1 if int_dato == mdy(09,11,2020)

replace avg_sun = 2.2 if int_dato == mdy(09,12,2020)

replace avg_sun = 0.6 if int_dato == mdy(09,13,2020)

replace avg_sun = 10.1 if int_dato == mdy(09,14,2020)

replace avg_sun = 8.9 if int_dato == mdy(09,15,2020)

replace avg_sun = 7.0 if int_dato == mdy(09,16,2020)

replace avg_sun = 10.9 if int_dato == mdy(09,17,2020)

replace avg_sun = 11.1 if int_dato == mdy(09,18,2020)

replace avg_sun = 6.6 if int_dato == mdy(09,19,2020)

replace avg_sun = 5.2 if int_dato == mdy(09,20,2020)

replace avg_sun = 5.3 if int_dato == mdy(09,21,2020)

replace avg_sun = 8.4 if int_dato == mdy(09,22,2020)

replace avg_sun = 5.8 if int_dato == mdy(09,23,2020)

replace avg_sun = 5.9 if int_dato == mdy(09,24,2020)

replace avg_sun = 0.2 if int_dato == mdy(09,25,2020)

replace avg_sun = 0.3 if int_dato == mdy(09,26,2020)

replace avg_sun = 3.3 if int_dato == mdy(09,27,2020)

replace avg_sun = 0.6 if int_dato == mdy(09,28,2020)

replace avg_sun = 3.6 if int_dato == mdy(09,29,2020)

replace avg_sun = 2.1 if int_dato == mdy(09,30,2020)

replace avg_sun = 4.7 if int_dato == mdy(11,03,2020)

replace avg_sun = 5.2 if int_dato == mdy(11,04,2020)

replace avg_sun = 0.3 if int_dato == mdy(11,05,2020)

replace avg_sun = 3.0 if int_dato == mdy(11,06,2020)

replace avg_sun = 1.6 if int_dato == mdy(11,07,2020)

replace avg_sun = 6.0 if int_dato == mdy(11,08,2020)

replace avg_sun = 0.0 if int_dato == mdy(11,09,2020)

replace avg_sun = 0.0 if int_dato == mdy(11,10,2020)

replace avg_sun = 0.0 if int_dato == mdy(11,11,2020)

replace avg_sun = 0.0 if int_dato == mdy(11,12,2020)

replace avg_sun = 2.2 if int_dato == mdy(11,13,2020)

replace avg_sun = 2.6 if int_dato == mdy(11,14,2020)

replace avg_sun = 2.6 if int_dato == mdy(11,15,2020)

replace avg_sun = 1.6 if int_dato == mdy(11,16,2020)

replace avg_sun = 0.0 if int_dato == mdy(11,17,2020)

replace avg_sun = 0.0 if int_dato == mdy(11,18,2020)

replace avg_sun = 3.0 if int_dato == mdy(11,19,2020)

replace avg_sun = 4.9 if int_dato == mdy(11,20,2020)

replace avg_sun = 0.0 if int_dato == mdy(11,21,2020)

replace avg_sun = 3.7 if int_dato == mdy(11,22,2020)

replace avg_sun = 4.4 if int_dato == mdy(11,23,2020)

replace avg_sun = 0.1 if int_dato == mdy(11,24,2020)

replace avg_sun = 1.6 if int_dato == mdy(11,25,2020)

replace avg_sun = 4.1 if int_dato == mdy(11,26,2020)

replace avg_sun = 4.7 if int_dato == mdy(11,27,2020)

replace avg_sun = 0.8 if int_dato == mdy(11,28,2020)

replace avg_sun = 0.1 if int_dato == mdy(11,29,2020)

replace avg_sun = 0.1 if int_dato == mdy(11,30,2020)

replace avg_sun = 3.6 if int_dato == mdy(03,02,2021)

replace avg_sun = 2.7 if int_dato == mdy(03,03,2021)

replace avg_sun = 6.4 if int_dato == mdy(03,04,2021)

replace avg_sun = 9.5 if int_dato == mdy(03,05,2021)

replace avg_sun = 3.5 if int_dato == mdy(03,06,2021)

replace avg_sun = 4.9 if int_dato == mdy(03,07,2021)

replace avg_sun = 9.8 if int_dato == mdy(03,08,2021)

replace avg_sun = 9.7 if int_dato == mdy(03,09,2021)

replace avg_sun = 2.9 if int_dato == mdy(03,10,2021)

replace avg_sun = 0.1 if int_dato == mdy(03,11,2021)

replace avg_sun = 1.6 if int_dato == mdy(03,12,2021)

replace avg_sun = 1.1 if int_dato == mdy(03,13,2021)

replace avg_sun = 3.6 if int_dato == mdy(03,14,2021)

replace avg_sun = 2.2 if int_dato == mdy(03,15,2021)

replace avg_sun = 8.2 if int_dato == mdy(03,16,2021)

replace avg_sun = 6.1 if int_dato == mdy(03,17,2021)

replace avg_sun = 7.2 if int_dato == mdy(03,18,2021)

replace avg_sun = 6.5 if int_dato == mdy(03,19,2021)

replace avg_sun = 0.9 if int_dato == mdy(03,20,2021)

replace avg_sun = 9.1 if int_dato == mdy(03,21,2021)

replace avg_sun = 3.3 if int_dato == mdy(03,22,2021)

replace avg_sun = 0.5 if int_dato == mdy(03,23,2021)

replace avg_sun = 1.8 if int_dato == mdy(03,24,2021)

replace avg_sun = 6.2 if int_dato == mdy(03,25,2021)

replace avg_sun = 2.6 if int_dato == mdy(03,26,2021)

replace avg_sun = 0.7 if int_dato == mdy(04,04,2021)

replace avg_sun = 5.5 if int_dato == mdy(04,05,2021)

replace avg_sun = 7.8 if int_dato == mdy(04,06,2021)

replace avg_sun = 5.5 if int_dato == mdy(04,07,2021)

replace avg_sun = 3.1 if int_dato == mdy(04,08,2021)

replace avg_sun = 5.9 if int_dato == mdy(04,09,2021)

replace avg_sun = 9.8 if int_dato == mdy(04,10,2021)

replace avg_sun = 0.1 if int_dato == mdy(04,11,2021)

replace avg_sun = 7.4 if int_dato == mdy(04,12,2021)

replace avg_sun = 7.9 if int_dato == mdy(04,13,2021)

replace avg_sun = 9.2 if int_dato == mdy(04,14,2021)

replace avg_sun = 9.4 if int_dato == mdy(04,15,2021)

replace avg_sun = 11.0 if int_dato == mdy(04,16,2021)

replace avg_sun = 7.5 if int_dato == mdy(04,17,2021)

replace avg_sun = 11.1 if int_dato == mdy(04,18,2021)

replace avg_sun = 13.2 if int_dato == mdy(04,19,2021)

replace avg_sun = 13.2 if int_dato == mdy(04,20,2021)

replace avg_sun = 8.7 if int_dato == mdy(04,21,2021)

replace avg_sun = 10.1 if int_dato == mdy(04,22,2021)

replace avg_sun = 9.6 if int_dato == mdy(04,23,2021)

replace avg_sun = 11.8 if int_dato == mdy(04,24,2021)

replace avg_sun = 1.4 if int_dato == mdy(09,13,2021)

replace avg_sun = 2.5 if int_dato == mdy(09,14,2021)

replace avg_sun = 0.3 if int_dato == mdy(09,15,2021)

replace avg_sun = 0.8 if int_dato == mdy(09,16,2021)

replace avg_sun = 0.2 if int_dato == mdy(09,17,2021)

replace avg_sun = 0.5 if int_dato == mdy(09,18,2021)

replace avg_sun = 1.4 if int_dato == mdy(09,19,2021)

replace avg_sun = 2.9 if int_dato == mdy(09,20,2021)

replace avg_sun = 1.7 if int_dato == mdy(09,21,2021)

replace avg_sun = 7.4 if int_dato == mdy(09,22,2021)

replace avg_sun = 1.2 if int_dato == mdy(09,23,2021)

replace avg_sun = 1.0 if int_dato == mdy(09,24,2021)

replace avg_sun = 6.7 if int_dato == mdy(09,25,2021)

replace avg_sun = 4.0 if int_dato == mdy(09,26,2021)

replace avg_sun = 0.5 if int_dato == mdy(09,27,2021)

replace avg_sun = 8.2 if int_dato == mdy(09,28,2021)

replace avg_sun = 2.5 if int_dato == mdy(09,29,2021)

replace avg_sun = 1.9 if int_dato == mdy(09,30,2021)

replace avg_sun = 1.0 if int_dato == mdy(10,01,2021)

replace avg_sun = 1.1 if int_dato == mdy(10,02,2021)

replace avg_sun = 0 if int_dato == mdy(10,03,2021)

replace avg_sun = 2.2 if int_dato == mdy(10,04,2021)

replace avg_sun = 2.2 if int_dato == mdy(10,05,2021)

replace avg_sun = 3.7 if int_dato == mdy(10,06,2021)

replace avg_sun = 8.1 if int_dato == mdy(10,07,2021)

replace avg_sun = 5.5 if int_dato == mdy(10,08,2021)

replace avg_sun = 5.0 if int_dato == mdy(10,09,2021)

replace avg_sun = 4.2 if int_dato == mdy(10,10,2021)

replace avg_sun = 5.3 if int_dato == mdy(10,11,2021)

replace avg_sun = 8.0 if int_dato == mdy(10,12,2021)

replace avg_sun = 4.2 if int_dato == mdy(10,13,2021)

replace avg_sun = 1.7 if int_dato == mdy(10,14,2021)

replace avg_sun = 6.1 if int_dato == mdy(10,15,2021)

replace avg_sun = 3.4 if int_dato == mdy(10,16,2021)

replace avg_sun = 2.1 if int_dato == mdy(10,17,2021)

replace avg_sun = 0.1 if int_dato == mdy(10,18,2021)

replace avg_sun = 0.1 if int_dato == mdy(10,19,2021)

replace avg_sun = 0.2 if int_dato == mdy(10,20,2021)

replace avg_sun = 2.9 if int_dato == mdy(10,21,2021)

replace avg_sun = 4.1 if int_dato == mdy(10,22,2021)

replace avg_sun = 5.8 if int_dato == mdy(10,23,2021)

replace avg_sun = 3.4 if int_dato == mdy(10,24,2021)

replace avg_sun = 2.7 if int_dato == mdy(10,25,2021)

replace avg_sun = 3.8 if int_dato == mdy(10,26,2021)

replace avg_sun = 0 if int_dato == mdy(10,27,2021)

replace avg_sun = 4.4 if int_dato == mdy(10,28,2021)

replace avg_sun = 6.6 if int_dato == mdy(10,29,2021)

replace avg_sun = 1.9 if int_dato == mdy(10,30,2021)

replace avg_sun = 3.0 if int_dato == mdy(10,31,2021)

replace avg_sun = 1.4 if int_dato == mdy(11,01,2021)

replace avg_sun = 3.0 if int_dato == mdy(11,02,2021)

replace avg_sun = 4.3 if int_dato == mdy(11,03,2021)

replace avg_sun = 0.9 if int_dato == mdy(11,04,2021)

replace avg_sun = 3.8 if int_dato == mdy(11,05,2021)

replace avg_sun = 0.4 if int_dato == mdy(11,06,2021)

replace avg_sun = 2.9 if int_dato == mdy(11,07,2021)

replace avg_sun = 0.8 if int_dato == mdy(11,08,2021)

replace avg_sun = 0.2 if int_dato == mdy(11,09,2021)

replace avg_sun = 0.5 if int_dato == mdy(11,10,2021)

replace avg_sun = 1.2 if int_dato == mdy(11,11,2021)

replace avg_sun = 0.8 if int_dato == mdy(11,12,2021)

replace avg_sun = 0 if int_dato == mdy(11,13,2021)

replace avg_sun = 0 if int_dato == mdy(11,14,2021)

replace avg_sun = 0 if int_dato == mdy(11,15,2021)

replace avg_sun = 0 if int_dato == mdy(11,16,2021)

replace avg_sun = 1.2 if int_dato == mdy(11,17,2021)

replace avg_sun = 0.1 if int_dato == mdy(11,18,2021)

replace avg_sun = 1.5 if int_dato == mdy(11,19,2021)

replace avg_sun = 0.2 if int_dato == mdy(11,20,2021)

replace avg_sun = 2.9 if int_dato == mdy(11,21,2021)

replace avg_sun = 4.6 if int_dato == mdy(11,22,2021)

replace avg_sun = 3.5 if int_dato == mdy(11,23,2021)

replace avg_sun = 0.7 if int_dato == mdy(11,24,2021)

replace avg_sun = 2.3 if int_dato == mdy(11,25,2021)

replace avg_sun = 1.9 if int_dato == mdy(11,26,2021)

replace avg_sun = 0.9 if int_dato == mdy(11,27,2021)

replace avg_sun = 0.2 if int_dato == mdy(11,28,2021)

replace avg_sun = 3.7 if int_dato == mdy(11,29,2021)

replace avg_sun = 1.7 if int_dato == mdy(11,30,2021)

replace avg_sun = 0 if int_dato == mdy(12,01,2021)

replace avg_sun = 3.4 if int_dato == mdy(12,02,2021)

replace avg_sun = 0.1 if int_dato == mdy(12,03,2021)

replace avg_sun = 0.1 if int_dato == mdy(12,04,2021)

replace avg_sun = 0.3 if int_dato == mdy(12,05,2021)

replace avg_sun = 1.3 if int_dato == mdy(12,06,2021)

replace avg_sun = 0.2 if int_dato == mdy(12,07,2021)

replace avg_sun = 0 if int_dato == mdy(12,08,2021)

replace avg_sun = 0 if int_dato == mdy(12,09,2021)

replace avg_sun = 0 if int_dato == mdy(12,10,2021)

replace avg_sun = 0.8 if int_dato == mdy(12,11,2021)

replace avg_sun = 0 if int_dato == mdy(12,12,2021)

replace avg_sun = 0 if int_dato == mdy(12,13,2021)

replace avg_sun = 2.5 if int_dato == mdy(12,14,2021)

replace avg_sun = 0.6 if int_dato == mdy(12,15,2021)

replace avg_sun = 5.5 if int_dato == mdy(12,16,2021)

replace avg_sun = 0.3 if int_dato == mdy(12,17,2021)

replace avg_sun = 3.1 if int_dato == mdy(12,18,2021)

replace avg_sun = 4.3 if int_dato == mdy(12,19,2021)

replace avg_sun = 5.5 if int_dato == mdy(12,20,2021)

replace avg_sun = 1.5 if int_dato == mdy(12,21,2021)

replace avg_sun = 0.6 if int_dato == mdy(12,22,2021)

replace avg_sun = 0 if int_dato == mdy(12,23,2021)

replace avg_sun = 2.4 if int_dato == mdy(12,24,2021)

replace avg_sun = 5.6 if int_dato == mdy(12,25,2021)

replace avg_sun = 5.0 if int_dato == mdy(12,26,2021)

replace avg_sun = 3.3 if int_dato == mdy(12,27,2021)

replace avg_sun = 0 if int_dato == mdy(12,28,2021)

replace avg_sun = 0.1 if int_dato == mdy(12,29,2021)

replace avg_sun = 0.2 if int_dato == mdy(12,30,2021)

}

replace avg_sun = 9.2 if int_dato == mdy(02,28,2022)

replace avg_sun = 2.4 if int_dato == mdy(03,01,2022)

replace avg_sun = 6.5 if int_dato == mdy(03,02,2022)

replace avg_sun = 7.5 if int_dato == mdy(03,03,2022)

replace avg_sun = 7.9 if int_dato == mdy(03,04,2022)

replace avg_sun = 5.7 if int_dato == mdy(03,05,2022)

replace avg_sun = 8.6 if int_dato == mdy(03,06,2022)

replace avg_sun = 9.6 if int_dato == mdy(03,07,2022)

replace avg_sun = 7.4 if int_dato == mdy(03,08,2022)

replace avg_sun = 6.5 if int_dato == mdy(03,09,2022)

replace avg_sun = 7.2 if int_dato == mdy(03,10,2022)

replace avg_sun = 10.2 if int_dato == mdy(03,11,2022)

replace avg_sun = 9.8 if int_dato == mdy(03,12,2022)

replace avg_sun = 9.6 if int_dato == mdy(03,13,2022)

replace avg_sun = 8.9 if int_dato == mdy(03,14,2022)

replace avg_sun = 3.8 if int_dato == mdy(03,15,2022)

replace avg_sun = 3.4 if int_dato == mdy(03,16,2022)

replace avg_sun = 0.6 if int_dato == mdy(03,17,2022)

replace avg_sun = 9.7 if int_dato == mdy(03,18,2022)

replace avg_sun = 10.6 if int_dato == mdy(03,19,2022)

replace avg_sun = 9.3 if int_dato == mdy(03,20,2022)

replace avg_sun = 8.9 if int_dato == mdy(03,21,2022)

replace avg_sun = 9.7 if int_dato == mdy(03,22,2022)

replace avg_sun = 10.0 if int_dato == mdy(03,23,2022)

replace avg_sun = 11.0 if int_dato == mdy(03,24,2022)

replace avg_sun = 10.0 if int_dato == mdy(03,25,2022)

replace avg_sun = 8.9 if int_dato == mdy(03,26,2022)

replace avg_sun = 1.1 if int_dato == mdy(04,04,2022)

replace avg_sun = 6.7 if int_dato == mdy(04,05,2022)

replace avg_sun = 0.4 if int_dato == mdy(04,06,2022)

replace avg_sun = 1.7 if int_dato == mdy(04,07,2022)

replace avg_sun = 6.9 if int_dato == mdy(04,08,2022)

replace avg_sun = 8.0 if int_dato == mdy(04,09,2022)

replace avg_sun = 8.6 if int_dato == mdy(04,10,2022)

replace avg_sun = 10.3 if int_dato == mdy(04,11,2022)

replace avg_sun = 7.1 if int_dato == mdy(04,12,2022)

replace avg_sun = 3.9 if int_dato == mdy(04,13,2022)

replace avg_sun = 0.6 if int_dato == mdy(04,14,2022)

replace avg_sun = 0.7 if int_dato == mdy(04,15,2022)

replace avg_sun = 11.3 if int_dato == mdy(04,16,2022)

replace avg_sun = 12.7 if int_dato == mdy(04,17,2022)

replace avg_sun = 12.9 if int_dato == mdy(04,18,2022)

preserve

collapse avg_sun avg_temp, by(int_dato)

foreach v in avg_sun avg_temp{

if "`v'"=="avg_sun" local name sun

if "`v'"=="avg_temp" local name temp

gen lag_`name' = `v'[_n-1]

}

keep int_dato lag_*

tempfile junk

save `junk'

restore

merge m:1 int_dato using `junk', nogen

gen month = month(int_dato)

**** Variable to identify less than 6 observations (to secure anonymity, cf. Statistics Denmark legislation)

gen count=0

sum int_dato

foreach d of numlist `r(min)'/`r(max)' {

sum WHO5 if int_dato==`d'

replace count = 1 if `r(N)'<6 & int_dato==`d'

}

**** Additional dataset for plotting outputs and temperature

preserve

collapse WHO5 WHO5_50 avg_temp avg_sun round count, by(int_dato)

rename int_dato date

format %tdDD-NN-CCYY date

export delimited using "$xxx\R\in\WHO5_Temp.csv", replace

restore

**** Respondents' age

tempvar birth

gen `birth'= foed_dag

format `birth' %td

tempvar surv

gen `surv'= int_dato

format `surv' %td

personage `birth' `surv', gen(age)

**** Age groups

gen age_groups = .

replace age_groups = 1 if age>=18 & age <=29

replace age_groups = 2 if age>=30 & age <=39

replace age_groups = 3 if age>=40 & age <=49

replace age_groups = 4 if age>=50 & age <=59

replace age_groups = 5 if age>=60 & age <=69

replace age_groups = 6 if age>=70 & age !=.

label define age_groups 1 "18-29" 2 "30-39" 3 "40-49" 4 "50-59" 5 "60-69" 6 "70-79"

label values age_groups age_groups

** Age groups

tab age_groups, gen(junk)

local low1 = 20

foreach v of numlist 1/`r(r)' {

if "`v'" == "1" local low 18

if "`v'" != "1" local low `low1'

if "`v'" == "1" local high 29

rename junk`v' Age`low'`high'

local low = `low' + 10

local high = `high' + 10

local low1 = `low1' + 10

}

//// Including data from SSI

preserve

use $yyy\data\COVID19_DK_May.dta, clear // read in SSI data

rename (date code_mun) (int_dato kom)

*** Dropping days where data is not updated

drop if int_dato>mdy(04,18,2022)

*** Daily total cases in DK

bys int_dato: egen cases_DK=sum(cases)

*** 7 days municpal moving average

rangestat (sum) cases, by(kom) int(int_dato -7 0)

rename cases_sum week_avg

*** 14 days municpal moving average

rangestat (sum) cases, by(kom) int(int_dato -14 0)

rename cases_sum twoweek_avg

*** Total citizens per municipality

bys kom: egen pop_kom = mean(total_citizens)

replace pop_kom = round(pop_kom)

label var pop_kom "Municipality's population"

*** Total population DK

bys int_dato: egen pop_DK = sum(pop_kom)

drop total_citizens

*** Infection rates

******* Daily DK

gen inf_rate_DK = (cases/pop_DK)*100000

label var inf_rate_DK "Daily incidence rate per 100k"

******* Daily per municipality

gen inf_rate_kom = (cases/pop_kom)*100000

label var inf_rate_kom "Municipality's daily incidence rate per 100k"

******* Weekly

gen inf_rate_kom_w = (week_avg/pop_kom)*100000

label var inf_rate_kom_w "Municipality's weekly incidence rate"

******* By-Weekly

gen inf_rate_kom_2w = (twoweek_avg/pop_kom)*100000

label var inf_rate_kom_2w "Municipality's by-weekly incidence rate"

tempfile junk

save `junk', replace

restore

******* Auxiliar dataset for plotting number of cases and incidences rates

preserve

use `junk', replace

collapse cases_DK,by(int_dato)

gen date = string(int_dato, "%tdCCYY-NN-DD")

order date cases_DK

drop int_dato

export delimited using "$xxx\R\in\cases.csv", replace

restore

/// Merging with data from SSI

sort int_dato kom

destring kom, replace

drop if kom == .

merge m:m int_dato kom using `junk', keep(3) nogen

*** Formating pnr var

format %15.0g pnr

save $xxx/do/do_for_submission/data/panel_unbalanced_march20_march22.dta, replace

***************************************************************************************

//////////////////////////////////////////////////////////////////////////////

// Title: Stress/Depression Across the COVID-19 Pandemic in Denmark

// Authors: BLINDED FOR REVIEW

// 02_descriptive00: This do-file makes Table 1 (Sample characteristics)

////////////////////////////////////////////////////////////////////////////////

/* Note: This dofile reports descriptive statistics about sample size, response rate, sample type and collection period */

putexcel set "$xxx/do/do_for_submission/output/Table1.xlsx", sheet(sample) modify

putexcel A2 = ("Survey Round")

putexcel B2 = ("Sample Size")

putexcel C2 = ("Response Rate")

putexcel D2 = ("Sample Type")

putexcel E2 = ("Collection period")

local row = 3

local num = 1

foreach samp in crosssection_March_2020 panel_July_2020 crosssection_September_2020 ///

panel_November_2020 crosssection_March_2021 panel_March_2021 ///

crosssection_September_2021 panel_November_2021 /// crosssection_March_2022 panel_March_2022 {

use $yyy/data/`samp'_stik.dta, clear

if ("`samp'"=="crosssection_March_2020" | ///

"`samp'"=="panel_July_2020" | ///

"`samp'"=="panel_November_2020" | ///

"`samp'"=="panel_March_2021" | ///

"`samp'"=="panel_November_2021" | ///

"`samp'"=="panel_March_2022") local samptype "Longitudinal"

else local samptype "Cross-sectional"

if "`samp'"=="crosssection_March_2020" local date "March 2 - April 13, 2020"

if "`samp'"=="panel_July_2020" local date "July 2 - August 01, 2020"

if "`samp'"=="crosssection_September_2020" local date "September 01 - September 30, 2020"

if "`samp'"=="panel_November_2020" local date "November 04 - November 30, 2020"

if "`samp'"=="crosssection_March_2021" local date "March 03 - April 24, 2021"

if "`samp'"=="panel_March_2021" local date "March 03 - April 24, 2021"

if "`samp'"=="crosssection_September_2021" local date "September 14 - November 26, 2021"

if "`samp'"=="panel_November_2021" local date "November 12 - December 30, 2021"

if "`samp'"=="crosssection_March_2022" local date "March 01 - April 18, 2022"

if "`samp'"=="panel_March_2022" local date "March 01 - April 18, 2022"

count

local total = r(N)

putexcel A`row' = `num'

putexcel B`row' = `total'

putexcel D`row' = ("`samptype'")

putexcel E`row' = ("`date'")

local row = `row'+1

local num = `num'+1

}

use $xxx/do/do_for_submission/data/panel_unbalanced_march20_march22.dta if WHO5!=., clear

qui sum wave

tab wave, matcell(freq)

putexcel F3 = matrix(freq)

foreach num of numlist 3/12 {

putexcel C`num' = formula(=100*(f`num'/B`num'))

}

tab int_dato if wave==1

***************************************************************************************

//////////////////////////////////////////////////////////////////////////////

// Title: Stress/Depression Across the COVID-19 Pandemic in Denmark

// Authors: BLINDED FOR REVIEW

// 03_analysis01: This do-file makes the analysis using a regression framework

////////////////////////////////////////////////////////////////////////////////

/* Note: This dofile makes the analysis of our variables of interest, and how these changed across survey waves. It also creates outputs for plotting our results. Also addresses possible seasonality issue */

/// Variables of interest

/* Note: Our dependent variables are the following:

* WHO5 ==> Measure scale from 0 to 100

* WHO5_50 ==> Measures risk of stress/depression, binary 0/1

/* When making the analyses, this dofile uses loops, and will refer to this variables in the local as "y", the survey wave as `r' and age group as `a' */

local data "$xxx/do/do_for_submission/data/panel_unbalanced_march20_march22.dta if WHO5!=., clear"

local data

/// Analysis

*Note: In this part we estimate the average on output for each age group

quietly {

foreach w in no yes {

use `data', clear

drop __00000V __00000W

if "`w'"=="yes" local weight "[aw=pervgt]"

else local weight

if "`w'"=="yes" local save "weight"

else local save "noweight"

foreach c in wocont wcont {

if "`c'"=="wcont" local season "lag_temp lag_sun"

else local season

local append replace

local table6 replace

foreach y in WHO5 WHO5_50 {

eststo m_`y'_`c': reg `y' i.bn.round#i.bn.age_groups `season' `weight', cl(pnr) nocons

if "`y'" == "WHO5" | "`y'" == "WHO5_50" {

if "`c'"=="wcont" local table6 append

else local table6 replace

margins i.bn.round#i.bn.age_groups, atmeans post

outreg2 using "$xxx\output\Table2_`y'_`save'_ci.xlsx", ci `table6' dec(3)

}

estimates restore m_`y'_`c'

margins i.bn.round#i.bn.age_groups, atmeans post

marginsplot , name(`y'_`c'_`save', replace)

foreach r of numlist 0/10 {

if "`r'"=="0" local round "0bn"

else local round `r'

foreach s of numlist 1/6 {

if "`s'"=="1" local age "1bn"

else local age `s'

gen b_`y'_r`r'_s`s'_`c' = _b[`round'.round#`age'.age_groups]

gen se_`y'_r`r'_s`s'_`c' = _se[`round'.round#`age'.age_groups]

}

}

}

}

keep round wave age_groups b_WHO5_* se_WHO5_* b_who5_* se_who5_*

collapse b_WHO5_* se_WHO5_* b_who5_* se_who5_* wave, by(age_groups round)

gen date = "3-01-2020" if round == 0

replace date = "3-22-2020" if round ==1

replace date = "7-15-2020" if round ==2

replace date = "9-15-2020" if round ==3

replace date = "11-15-2020" if round ==4

replace date = "3-01-2021" if round ==5

replace date = "3-30-2021" if round ==6

replace date = "9-14-2021" if round == 7

replace date = "11-12-2021" if round == 8

replace date = "3-01-2022" if round == 9

replace date = "3-30-2022" if round == 10

drop if round==.

foreach y in WHO5 WHO5_50 {

if "`y'" == "WHO5" local v "who5"

if "`y'" == "WHO5_50" local v "who5_50"

foreach c in wocont wcont {

gen b_`v'_`c'= .

gen se_`v'_`c'=.

foreach r of numlist 0/10 {

foreach s of numlist 1/6 {

replace b_`v'_`c'= b_`y'_r`r'_s`s'_`c' if round==`r' & age_groups==`s'

replace se_`v'_`c'= se_`y'_r`r'_s`s'_`c' if round==`r' & age_groups==`s'

}

}

}

}

keep age_groups round wave date b_who5_wocont se_who5_wocont ///

b_who5_wcont se_who5_wcont b_who5_50_wocont ///

se_who5_50_wocont b_who5_50_wcont se_who5_50_wcont

gen source = "cross-sectional" if round == 3 | round == 5 | round == 7 | round == 9

replace source = "panel" if round == 0 | round == 1 | round == 2 | round == 4 | round == 6 | ///

round == 8 | round == 10

sort round

drop if round==.

export delimited using "$xxx\R\in\graph1_`save'.csv", replace

}

}

***************************************************************************************

//////////////////////////////////////////////////////////////////////////////

// Title: Stress/Depression Across the COVID-19 Pandemic in Denmark

// Authors: BLINDED FOR REVIEW

// 03_analysis02: This do-file makes the anlysis in a regression framework

////////////////////////////////////////////////////////////////////////////////

/* Note: This dofile makes the analysis of our variables of interest, and how these changed across waves.

It also creates outputs for plotting our results. Also addresses seasonality issue */

/// Variables of interest

/* Note: Our dependent variables are the following:

* WHO5 ==> Measure stress, scale from 0 to 100

* WHO5_50 ==> Measures risk of severe depression, binary 0/1

When making the analyses, this dofile uses loops, and will refer to this variables in the local as "y", the survey wave as `r', and age group as `a'

*/

local data "$xxx/do/do_for_submission/data/panel_unbalanced_march20_march22.dta if WHO5!=., clear"

local data

//// Graphs and Tables

/*Note: In this part we estimate the average on output living arrangements and respondent's gender */

quietly {

foreach w in yes no {

preserve

use `data', clear

drop __00000V __00000W

if "`w'"=="yes" local weight "[aw=pervgt]"

else local weight

if "`w'"=="yes" local save "weight"

else local save "noweight"

foreach c in wocont wcont {

if "`c'"=="wcont" local season "lag_temp lag_sun"

else local season

local append replace

local table8 replace

foreach y in WHO5 WHO5_50 {

eststo m_`y'_`c': reg `y' ///

i.bn.round##(i.bn.koen#i.bn.Children) ///

`season' `weight', cl(pnr) nocons

if "`y'" == "WHO5" | "`y'" == "WHO5_50" {

if "`c'"=="wcont" local table8 append

else local table8 replace

margins i.bn.round#(i.bn.koen#i.bn.Children), atmeans post

outreg2 using "$xxx\output\Table3_`y'_`save'_ci.xlsx", ci `table8' dec(3)

}

estimates restore m_`y'_`c'

margins i.bn.round#(i.bn.koen#i.bn.Children), atmeans post

marginsplot , name(`y'_`c'_`save', replace)

foreach r of numlist 0/10 {

if "`r'"=="0" local round "0bn"

else local round `r'

foreach s of numlist 1/2 {

if "`s'"=="1" local sex "1bn"

else local sex `s'

foreach ch of numlist 0/1 {

if "`ch'"=="0" local child "0bn"

else local child `ch'

gen b_`y'_r`r'_s`s'_ch`ch'_`c' = _b[`r'bn.round#`s'.koen#`ch'.Children]

gen se_`y'_r`r'_s`s'_ch`ch'_`c' = _se[`r'bn.round#`s'.koen#`ch'.Children]

}

}

}

}

}

tempfile junk

save `junk', replace

restore

preserve

use `junk', clear

keep round wave koen Children b_WHO5_* se_WHO5_* b_who5_* se_who5_*

collapse b_WHO5_* se_WHO5_* b_who5_* se_who5_* wave, by(round koen Children)

gen date = "3-01-2020" if round == 0

replace date = "3-22-2020" if round ==1

replace date = "7-15-2020" if round ==2

replace date = "9-15-2020" if round ==3

replace date = "11-15-2020" if round ==4

replace date = "3-01-2021" if round ==5

replace date = "3-30-2021" if round ==6

replace date = "9-14-2021" if round == 7

replace date = "11-12-2021" if round == 8

replace date = "3-01-2022" if round == 9

replace date = "3-30-2022" if round == 10

drop if round==.

foreach y in WHO5 WHO5_50 {

if "`y'" == "WHO5" local v "who5"

if "`y'" == "WHO5_50" local v "who5_50"

if "`y'" == "who5_bl" local v "who5_bl"

if "`y'" == "who5_50_bl" local v "who5_50_bl"

foreach c in wocont wcont {

gen b_`v'_`c'= .

gen se_`v'_`c'=.

foreach r of numlist 0/10 {

foreach s of numlist 1/2 {

foreach ch of numlist 0/1 {

replace b_`v'_`c'= b_`y'_r`r'_s`s'_ch`ch'_`c' if round==`r' & koen==`s' & Children==`ch'

replace se_`v'_`c'= se_`y'_r`r'_s`s'_ch`ch'_`c' if round==`r' & koen==`s' & Children==`ch'

}

}

}

}

}

keep koen Children round wave date b_who5_wocont se_who5_wocont b_who5_wcont ///

se_who5_wcont b_who5_50_wocont se_who5_50_wocont b_who5_50_wcont se_who5_50_wcont

rename koen sex

label values sex .

label define sex 1 "Male" 2 "Female"

label values sex sex

foreach v in b_who5_wocont b_who5_wcont b_who5_50_wocont b_who5_50_wcont {

replace `v'=. if `v'==0

}

export delimited using "$xxx\R\in\graph2_`save'.csv", replace

restore

}

}

***************************************************************************************

//////////////////////////////////////////////////////////////////////////////

// Title: Stress/Depression Across the COVID-19 Pandemic in Denmark

// Authors: BLINDED FOR REVIEW

// 03_analysis: This do-file makes the analysis using Binscatter

////////////////////////////////////////////////////////////////////////////////

/* Note: This dofile uses a binscatter analysis to analyze the importance of local infection rates.

The dofile analyzes our variables of interest, and how these changed across waves and addresses seasonality issue and focuses on variation in local infection rates. */

/* Note: Our dependent variables are the following:

* WHO5 ==> Measure stress, scale from 0 to 100

* WHO5_50 ==> Measures risk of severe depression, binary 0/1

When making the analyses, this dofile uses loops, and will refer to this variables in the local as "y", the survey wave as `r', and age group as `a'

*/

/// Opening data

use $xxx/do/do_for_submission/data/panel_unbalanced_march20_march22.dta if WHO5!=., clear

drop __00000V __00000W

cd $xxx

/// Analysis

*** Generate logarithm of two weeks moving average infection rate

gen log_infrate = log(inf_rate_kom_2w)

label var log_infrate "Log Municipal infection rate"

rename (WHO5 WHO5_50) (who5 who5_50)

**** Generating a new scheme

grstyle init xxx, replace

grstyle set mesh, horizontal compact minor

grstyle linewidth major_grid thin

grstyle set legend 6, nobox

grstyle set color viridis, n(9)

grstyle set symbolsize vsmall

grstyle linewidth plineplot thin

grstyle legend_key_xsize small

grstyle legend_key_ysize small

grstyle numstyle legend_cols 9

grstyle type

set scheme xxx

**** Binscatter analysis

foreach y in who5_50 who5 {

foreach x in inf_rate_kom_2w log_infrate {

if "`y'" == "who5" local yaxis "WHO5"

if "`y'" == "who5_50" local yaxis "Pr(WHO5<50)"

if "`x'" == "inf_rate_kom_2w" local xaxis "Two weeks average incidence rate"

if "`x'" == "log_infrate" local xaxis "Log(two weeks average incidence rate)"

if ("`y'" == "who5" & "`x'" == "inf_rate_kom_2w" ) local store "binscatter_WHO5_rate"

if ("`y'" == "who5" & "`x'" == "log_infrate" ) local store "binscatter_WHO5_log"

if ("`y'" == "who5_50" & "`x'" == "inf_rate_kom_2w") local store "binscatter_WHO5_50_rate"

if ("`y'" == "who5_50" & "`x'" == "log_infrate") local store "binscatter_WHO5_50_log"

foreach r of numlist 0/10 {

preserve

qui eststo `y'_`x'_age`r': reg `y' `x' ibn.age_groups lag_temp lag_sun if round==`r' [aw=pervgt], nocons

binscatter `y' `x' if round==`r' [aw=pervgt], controls(i.age_groups lag_temp lag_sun) savedata(binscatter/`y'_`x'_age`r') replace nodraw

clear

do binscatter/`y'_`x'_age`r'

tempfile `y'_`x'_age`r'

save ``y'_`x'_age`r'', replace

restore

preserve

qui eststo `y'_`x'_child`r': reg `y' `x' i.bn.koen#i.bn.Children lag_temp lag_sun if round==`r' [aw=pervgt], nocons

binscatter `y' `x' if round==`r' [aw=pervgt], controls(i.bn.koen#i.bn.Children lag_temp lag_sun) savedata(binscatter/`y'_`x'_child`r') replace nodraw

clear

do binscatter/`y'_`x'_child`r'

tempfile `y'_`x'_child`r'

save ``y'_`x'_child`r'', replace

restore

}

foreach c in age child {

foreach r of numlist 0/10 {

append using ``y'_`x'_`c'`r'', gen(`y'_bin_`c'_r`r')

}

}

twoway lfit `y' `x' if `y'_bin_age_r0==1, lp(solid) lc(purple) || scatter `y' `x' if `y'_bin_age_r0==1, mc(purple) msize(tiny) ///

|| lfit `y' `x' if `y'_bin_age_r1==1, lp(solid) lc(navy) || scatter `y' `x' if `y'_bin_age_r1==1, mc(navy) msize(tiny) ///

|| lfit `y' `x' if `y'_bin_age_r2==1, lp(solid) lc(edkblue) || scatter `y' `x' if `y'_bin_age_r2==1, mc(edkblue) msize(tiny) ///

|| lfit `y' `x' if `y'_bin_age_r3==1, lp(solid) lc(ebblue) || scatter `y' `x' if `y'_bin_age_r3==1, mc(ebblue) msize(tiny) ///

|| lfit `y' `x' if `y'_bin_age_r4==1, lp(solid) lc(emerald) || scatter `y' `x' if `y'_bin_age_r4==1, mc(emerald) msize(tiny) ///

|| lfit `y' `x' if `y'_bin_age_r5==1, lp(solid) lc(midgreen) || scatter `y' `x' if `y'_bin_age_r5==1, mc(midgreen) msize(tiny) ///

|| lfit `y' `x' if `y'_bin_age_r6==1, lp(solid) lc(mint) || scatter `y' `x' if `y'_bin_age_r6==1, mc(mint) msize(tiny) ///

|| lfit `y' `x' if `y'_bin_age_r7==1, lp(solid) lc(sandb) || scatter `y' `x' if `y'_bin_age_r7==1, mc(sandb) msize(tiny) ///

|| lfit `y' `x' if `y'_bin_age_r8==1, lp(solid) lc(yellow) || scatter `y' `x' if `y'_bin_age_r8==1, mc(yellow) msize(tiny) ///

|| lfit `y' `x' if `y'_bin_age_r9==1, lp(solid) lc(red) || scatter `y' `x' if `y'_bin_age_r9==1, mc(red) msize(tiny) ///

|| lfit `y' `x' if `y'_bin_age_r10==1, lp(solid) lc(green) || scatter `y' `x' if `y'_bin_age_r10==1, mc(green) msize(tiny) ///

xtitle("", size(small)) ytitle(`yaxis', size(small)) ///

legend(off) t1title("A) Controlling for age groups", size(small) just(left) bexpand) xscale(range(-1 8.9)) name(age, replace)

twoway lfit `y' `x' if `y'_bin_child_r0==1, lp(solid) lc(purple) || scatter `y' `x' if `y'_bin_child_r0==1, mc(purple) msize(tiny) ///

|| lfit `y' `x' if `y'_bin_child_r1==1, lp(solid) lc(navy) || scatter `y' `x' if `y'_bin_child_r1==1, mc(navy) msize(tiny) ///

|| lfit `y' `x' if `y'_bin_child_r2==1, lp(solid) lc(edkblue) || scatter `y' `x' if `y'_bin_child_r2==1, mc(edkblue) msize(tiny) ///

|| lfit `y' `x' if `y'_bin_child_r3==1, lp(solid) lc(ebblue) || scatter `y' `x' if `y'_bin_child_r3==1, mc(ebblue) msize(tiny) ///

|| lfit `y' `x' if `y'_bin_child_r4==1, lp(solid) lc(emerald) || scatter `y' `x' if `y'_bin_child_r4==1, mc(emerald) msize(tiny) ///

|| lfit `y' `x' if `y'_bin_child_r5==1, lp(solid) lc(midgreen) || scatter `y' `x' if `y'_bin_child_r5==1, mc(midgreen) msize(tiny) ///

|| lfit `y' `x' if `y'_bin_child_r6==1, lp(solid) lc(mint) || scatter `y' `x' if `y'_bin_child_r6==1, mc(mint) msize(tiny) ///

|| lfit `y' `x' if `y'_bin_child_r7==1, lp(solid) lc(sandb) || scatter `y' `x' if `y'_bin_child_r7==1, mc(sandb) msize(tiny) ///

|| lfit `y' `x' if `y'_bin_child_r8==1, lp(solid) lc(yellow) || scatter `y' `x' if `y'_bin_child_r8==1, mc(yellow) msize(tiny) ///

|| lfit `y' `x' if `y'_bin_child_r9==1, lp(solid) lc(red) || scatter `y' `x' if `y'_bin_child_r9==1, mc(red) msize(tiny) ///

|| lfit `y' `x' if `y'_bin_child_r10==1, lp(solid) lc(green) || scatter `y' `x' if `y'_bin_child_r10==1, mc(green) msize(tiny) ///

xtitle(`xaxis', size(small)) ytitle(`yaxis', size(small)) ///

legend(lab(1 "Mar-2020*") lab(3 "Mar-2020") lab(5 "Jul-2020") ///

lab(7 "Sept-2020") lab(9 "Nov-2020") lab(11 "Mar-2021") ///

lab(13 "Mar-2021") lab(15 "Sept-2021") lab(17 "Nov-2021") ///

lab(19 "Mar-2022") lab(21 "Mar-2022") order(1 3 5 7 9 11 13 15 17 19 21) rows(1)) ///

t1title("B) Controlling for gender and living arrangements", size(small) just(left) bexpand) ///

xscale(range(-1 8.9)) name(child, replace)

gr combine age child, rows(2) name(`y'_`x', replace)

gr display, xsize(30) ysize(15)

gr export "$xxx\binscatter\graph_`store'.png", replace

}

}

*** Export binscatter results

foreach y in who5_50 who5 {

foreach v in age child {

if "`v'" == "age" local title "age_groups"

else local title "child"

esttab `y'_log_infrate_`v'0 `y'_log_infrate_`v'1 ///

`y'_log_infrate_`v'2 `y'_log_infrate_`v'3 ///

`y'_log_infrate_`v'4 `y'_log_infrate_`v'5 ///

`y'_log_infrate_`v'6 `y'_log_infrate_`v'7 ///

`y'_log_infrate_`v'8 `y'_log_infrate_`v'9 ///

`y'_log_infrate_`v'10 ///

using $xxx/binscatter/`y'_`title'.rtf, se replace mtitles

}

}

***************************************************************************************

//////////////////////////////////////////////////////////////////////////////

// Title: Stress/Depression Across the COVID-19 Pandemic in Denmark

// Authors: BLINDED FOR REVIEW

// r&r: This do-file makes tables for the r&r

////////////////////////////////////////////////////////////////////////////////

/* Note: This dofile creates the tables included after the r&r*/

clear all

set graphics on

version 16

use $xxx/data/panel_unbalanced_march20_march22.dta if WHO5!=. & WSAS!=., clear

drop __00000V __00000W

*** Estimations including intercept

*** Probability of suffering from stress anxiety by age groups

foreach num of numlist 0/10 {

eststo model_r`num': reg WHO5_50 ib1.aldergrp lag_temp lag_sun [aw=pervgt] if round==`num', cl(pnr)

}

esttab model_r0 model_r1 model_r2 model_r3 model_r4 model_r5 model_r6 model_r7 model_r8 model_r9 model_r10 using $xxx\output\WHO5_50_round_intercept.rtf, ci(2) parentheses compress label keep(1.aldergrp 2.aldergrp 3.aldergrp 4.aldergrp 5.aldergrp 6.aldergrp _cons) replace

*** Probability of suffering from stress anxiety by gender and living arrangements

***** Labeling variables

label define Children 0 "No children" 1 "Children"

label values Children Children

label values koen .

label define koen 1 "Men" 2 "Women"

label values koen koen

foreach r of numlist 0/10 {

eststo ch_r`r': reg WHO5_50 i.b1.koen#i.b0.Children lag_temp lag_sun [aw=pervgt] if round==`r', cl(pnr)

}

esttab ch_r0 ch_r1 ch_r2 ch_r3 ch_r4 ch_r5 ch_r6 ch_r7 ch_r8 ch_r9 ch_r10 using $xxx\output\WHO5_50_children_gender_intercept.rtf, ci(2) parentheses compress label interaction("x") keep(1.koen#0.Children 1.koen#1.Children 2.koen#0.Children 2.koen#1.Children _cons) replace

*** Tables comparing survey waves and population

*** Comparing survey rounds (panel)

foreach comp in 1 2 3 4 {

disp in red "Comparison `comp'"

use $xxx/data/sample_pop_comparison.dta, clear

if "`comp'"== "1" /*all rounds vs population*/ {

local samp "round"

local num_start "0"

local cond "population == 1"

local cond1 "of numlist 0/10"

local cond2 "of numlist 1/10"

local start_append "0"

local save "Table_rounds_vs_population"

}

if "`comp'"== "2" /*crossectional survey vs population*/ {

local samp "wave"

local num_start "1"

local cond "population == 1"

local cond1 "in 1 3 5 7 9"

local cond2 "in 3 5 7 9"

local start_append "1"

local save "Table_cross_sec_vs_population"

}

if "`comp'"== "3" /*longitudinal surveys vs pre-pandemic*/ {

local samp "round"

local num_start "2"

local cond "round == 0"

local cond1 "in 2 4 6 8 10"

local cond2 "in 4 6 8 10"

local start_append "2"

local save "Table_panel_vs_prepandemic"

}

if "`comp'"== "4" /*Pre-pandemic vs post first lockdown*/ {

local samp "round"

local num_start "0"

local cond "round == 0"

local cond1 "in 1"

local cond2 "in 1"

local start_append "1"

local save "Table_prepandemic_vs_firstlockdown"

}

*** Starting loop

foreach r `cond1' {

preserve

keep if `samp' == `r' | `cond'

tempvar dum

gen `dum'= inlist(`samp',`r')

tempfile junk

save `junk', replace

tab `samp' `dum'

restore

local n=0

foreach v in Female ///

Age1829 Age3039 Age4049 Age5059 Age6069 Age7079 ///

Couple ///

Children {

preserve

use `junk', clear

display in red "************** Round `r'-- `v'"

ttest `v', by(`dum')

ge Variable="`v'"

ge Survey=r(mu_1)

ge Population=r(mu_2)

ge sd_Survey=r(sd_1)

gen N_Survey=r(N_1)

ge sd_Population=r(sd_2)

qui reg `v' `dum' [aw=pervgt]

local tstat = _b[`dum']/_se[`dum']

local ppop= (2*ttail(e(df_r), abs(_b[`dum']/_se[`dum'])))

ge t=`tstat'

ge p=`ppop'

keep if _n==1

replace wave=`r'

keep Variable Survey sd_Survey Population sd_Population t p `samp' N_Survey

order Variable Survey sd_Survey Population sd_Population t p `samp' N_Survey

ge n=`n'

if `n'>1 append using `SurveyPopTest'

local n=`n'+1

sort n

drop n

tempfile SurveyPopTest

save `SurveyPopTest', replace

restore

}

qui su `samp' if `samp'==`num_start'

local Ns=r(N)

qui su `samp' if `samp'==`r'

local Np=r(N)

preserve

use `SurveyPopTest', clear

*ge N_Survey=`Ns'

ge N_Population=`Np'

tempfile round_`r'

save `round_`r'', replace

restore

}

use `round_`start_append'', clear

if "`comp'"<= "4" {

foreach r `cond2' {

append using `round_`r''

}

}

order Variable Survey sd_Survey Population sd_Population t p `samp' N_Survey

tempfile table2

save `table2', replace

use `table2', clear

export excel using $xxx/output/`save'.xlsx, firstrow(variables) replace

}

< end of do-files >
